# The impact of de novo lipogenesis on predicting survival and clinical therapy: an exploration based on a multigene prognostic model in hepatocellular carcinoma

**DOI:** 10.1186/s12967-025-06704-y

**Published:** 2025-06-18

**Authors:** Xin Zhou, Guangzu Cui, Erya Hu, Xinwen Wang, Diya Tang, Xiangyang Zhang, Jiayao Ma, Yin Li, Haicong Liu, Qingping Peng, Ying Han, Yihong Chen, Shan Zeng, Yan Zhang, Hong Shen

**Affiliations:** 1 Department of Oncology, Xiangya Hospital, Central South University, Changsha, Hunan 410008 China; 2 National Clinical Research Center for Geriatric Disorders, Xiangya Hospital, Central South University, Changsha, Hunan 410008 China; 3 Department of Oncology, Yueyang People’s Hospital, Yueyang Hospital Affiliated to Hunan Normal University, Yueyang, Hunan 414000 China

**Keywords:** De novo lipogenesis, Multigene prognostic model, RNA-seq, Single-cell RNA-seq, Clinical therapy

## Abstract

**Background:**

Hepatocellular carcinoma (HCC) ranks among the most aggressive malignancies worldwide, with poor outcomes attributed to delayed diagnosis and therapeutic limitations. Emerging evidence suggests that de novo lipogenesis (DNL) plays a crucial role in HCC progression and its interaction with the immune microenvironment.

**Methods:**

We systematically analyzed DNL-related gene expression profiles from TCGA, GEO, ICGC-LIRI datasets, and our Xiangya HCC cohort (*n* = 106) to construct a prognostic risk model. Through LASSO-Cox regression analysis, we identified six signature genes (G6PD, LCAT, SERPINE1, SOAT2, CYP2C9, and UGT1A10) that effectively stratified patients into distinct risk groups. We evaluated clinical characteristics, immune cell infiltration patterns, and differential therapeutic responses between high-risk and low-risk groups. Comprehensive validation included immunohistochemical analysis and Western blotting to assess expression levels of key model genes, along with multiplex immunofluorescence staining and single-cell RNA sequencing(scRNA-seq) to characterize immune microenvironmental differences between risk groups.

**Results:**

We successfully established a robust six-gene prognostic signature (G6PD, LCAT, SERPINE1, SOAT2, CYP2C9, and UGT1A10) based on de novo lipogenesis pathways, which demonstrated excellent predictive performance (AUC: 0.78–0.82). The model revealed significant differences in immune infiltration patterns between risk groups, with the high-risk group exhibiting immunosuppressive characteristics characterized by increased Treg cell infiltration, while the low-risk group showed greater NK cell retention. Integrated scRNA-seq and our cohort validation further demonstrated that high-risk scores were associated with poorer response to immunotherapy but greater sensitivity to targeted therapies. These findings suggest that de novo lipogenesis-mediated immune evasion contributes to therapy resistance and worse prognosis in high-risk HCC patients, whereas low-risk HCC patients maintain an immunologically active microenvironment more amenable to immunotherapy.

**Conclusions:**

This study provided a novel prognostic model for HCC, incorporating 6 representative DNLs. The model demonstrated the potential for predicting HCC prognosis and highlighted the involvement of immune cell infiltration and the association between risk scores and clinical therapy. Validation of model genes further supported the association between de novo lipogenesis and HCC development.

**Supplementary Information:**

The online version contains supplementary material available at 10.1186/s12967-025-06704-y.

## Introduction

Primary liver cancer is a malignancy with high incidence and mortality rates. According to the global cancer statistics (2022), it ranks as the sixth most commonly diagnosed malignancy worldwide and the third leading cause of cancer-related deaths, with HCC accounting for 80% of cases, we need to strengthen global research collaboration on liver cancer to improve patient health outcomes [1–3]. Despite significant advances in genomics, immunology and molecular biology that have deepened our understanding of HCC pathogenesis and led to breakthroughs in targeted therapies and immunotherapies [4], the poor prognosis and strong drug resistance are still major challenges faced in clinical practice, with a five-year survival rate of merely 15% [5]. Therefore, there is an urgent need to find more reliable biomarkers to establish a prognostic risk model to predict the prognosis of patients with liver cancer and explore more potential therapeutic targets. Notably, accumulating evidence suggests that targeting DNL metabolic pathways may improve the efficacy of tumor treatment. However, there is currently little research on DNL-related characteristic genes in HCC patients.

Under physiological conditions, cells convert non-lipid carbon sources into lipids through the process known as de novo lipogenesis (DNL), which helps the body store energy. The fatty acids and triglycerides synthesized via the DNL pathway maintain energy balance and are also essential components of cell membranes. However, in pathological states, DNL provides necessary energy for tumor cells by synthesizing lipids to support their rapid proliferation, and it is closely related to tumor growth and metastasis [6, 7]. Numerous studies have shown that DNL is significantly increased in HCC, where dysregulated lipogenesis plays a pivotal regulatory role in hepatocarcinogenesis and disease progression [8, 9]. Sterol regulatory element-binding proteins (SREBPs) function as master regulators of the DNL pathway, exerting control over the expression of key metabolic enzymes including ATP citrate lyase (ACLY), acetyl-CoA carboxylase (ACAC), fatty acid synthase (FASN), and stearoyl-CoA desaturase 1 (SCD1) [10, 11]. Notably, pharmacological inhibition of SREBPs and their downstream metabolic enzymes has been shown to significantly suppress HCC development [12, 13]. The oncogenic activation of DNL also orchestrates an immunosuppressive tumor microenvironment in HCC, characterized by high levels of neutrophils associated with poor prognosis in patients [14]. In ovarian cancer, FASN overexpression compromises dendritic cell (DC) functionality, leading to T cell dysfunction [15]. Preclinical studies have confirmed that combining FASN inhibitors with anti-PD-L1 antibodies significantly suppresses tumor growth [16], while SCD1 inhibition not only enhances CD8 T cell infiltration but may also improve response to PD-1 blockade therapy [17]. These findings collectively demonstrate that DNL contributes not only closely related to tumor development but also modulates tumor immune microenvironment characteristics and immunotherapy efficacy.

In summary, we constructed a novel risk model based on six DNL-related genes (G6PD, LCAT, SERPINE1, SOAT2, CYP2C9, and UGT1A10) that demonstrates robust performance in prognostic stratification, immune cell infiltration, and immunotherapy response prediction in HCC patients. Additionally, we established a real-world Xiangya HCC cohort as a validation set to assess the treatment responses of high-risk and low-risk patients, finding that high-risk patients are more sensitive to targeted therapies, while low-risk patients are more sensitive to immunotherapy. Overall, These findings establish this DNL-based model not only as a reliable prognostic biomarker but also as a valuable tool for guiding personalized treatment decisions in clinical practice.

## Methods

### Data collection and clinical sample

RNA-seq data and the clinical information for 427 HCC samples were downloaded from the GDC date portal (https://portal.gdc.cancer.gov/). 14 samples were excluded due to not a primary tumor or lack prognostic clinical information. In total, 413 samples, comprising 363 tumor samples and 50 normal samples, were analyzed in the present study. Three validation cohorts, GSE14520(https://www.ncbi.nlm.nih.gov/), ICGC-LIRI cohort(https://xenabrowser.net), and Xiangya HCC cohort, were applied in the present study. Fresh tumor tissues were collected from 106 patients with HCC pathologically diagnosed at Xiangya Hospital of Central South University, with immediate snap-freezing in liquid nitrogen followed by RNA sequencing. Postoperative treatment outcomes were evaluated in 62 patients receiving either immune checkpoint blockade (ICB, *n* = 29) or tyrosine kinase inhibitors (TKIs, *n* = 33), with radiographic response assessed per mRECIST criteria [18]. Additionally, scRNA-seq (10x Genomics platform) was performed on tumor tissues from 8 representative cases. This study was approved by Ethics Committees of Xiangya Hospital. The list of de novo lipogenesis-related genes was collected from GeneCards (https://www.genecards.org/) [19] (Additional file 1: Table S1).

### Identification of differentially expressed genes (DEGs)

The R package “DEseq2” [20] was used to generate DEGs between normal and tumor samples or between high-risk and low-risk groups in the training set.|log_2_FoldChange|> 1.5 and adjusted *P* < 0.05 were the criteria for defining DEGs. Volcano plots were generated using the “ggplot2” package and “cowplot” package in R to visualize the DEGs, and Venn diagrams were used to display the intersection genes of the DEGs group with de novo lipogenesis-related genes.

### Construction and validation of prognostic de novo lipogenesis‑related risk score signature

Through a comprehensive analytical approach incorporating univariate Cox regression, LASSO regression, and multivariate Cox regression analyses, we systematically identified de novo lipogenesis-related prognostic genes and constructed a novel risk signature [21]. We utilized the R package “glmnet” to perform LASSO regression for variable selection, with parameters set to their default values. The optimal lambda (λ) value was selected through cross-validation. Following LASSO selection, multivariate Cox proportional hazards regression analysis was employed to further evaluate the retained genes. Genes with a p-value < 0.05 were considered statistically significant and included in the final model. The risk score for each patient with HCC in both training and validation cohorts was computed based on the following formula: Risk score = 0.218*G6PD − 0.222*LCAT + 0.142* SREPINE1 + 0.156*UGT1A10–0.255CYP2C9 + 0.237SOAT2. Using the median risk score as the cutoff, patients in both training and validation cohorts were stratified into high- and low-risk groups. Kaplan-Meier (K-M) survival curves were drawn using the “survival” and “survminer” R packages to compare the survival differences between the two groups. ROC curves, risk plots, and C-index (consistency index) were used to test the predictive effect of prognostic indicators in HCC patients.

### Functional enrichment analysis

In order to compare the differences of the molecular function and the enrichment pathway between high-risk group and low-risk group, the “clusterProfiler”, “org.Hs.eg.db”, and “Tidyverse” packages in R software were used to perform Gene Ontology (GO) analysis and Kyoto Encyclopedia of Genes and Genomes (KEGG) analysis for DEGs. The target gene sets in Gene Set Enrichment Analysis (GSEA) were obtained from Human Molecular Signatures Database (MSigDB) (https://www.gsea-msigdb.org/gsea/msigdb/collections.jsp) [22], and only gene sets with adjusted *P* value < 0.05 and q value < 0.25 were regarded to be statistically significant.

### Cluster analysis

Based on the six de novo lipogenesis-related signature genes, we performed consensus clustering of HCC samples from the TCGA-LIHC cohort using the “ConsensusClusterPlus” R package [23]. The optimal number of clusters was determined by selecting the K value that minimizes the within-cluster sum of squares, and a K value of 2 was considered ideal because the inter-group correlation was low while the intra-group correlation was high. To visualize the metabolic heterogeneity patterns, we employed both principal component analysis (PCA) for linear dimensionality reduction and t-distributed stochastic neighbor embedding (t-SNE) for nonlinear visualization. The CIBERSORT algorithm [24] was used to calculate the expression of 22 tumor-infiltrating immune cells in two clusters and was displayed as box plots. We compared the differences in survival rates between clusters by the K-M curve and log-rank test, and the differences in clinical characteristics (including age, sex, TNM stage) between the clusters were illustrated by heatmaps and bar charts.

### Mutation analysis

The mutation data obtained from the cBioportal database (https://www.cbioportal.org/) was analyzed by the “maftools” package of R [25], in order to examine the differences in TMB between the high-risk and low-risk HCC groups and to visualize the mutation landscape of tumor cell mutant genes in the two groups.

### Tumor microenvironment and immunotherapy response

The “IBOR” package of R was used to analyze the tumor microenvironment of HCC patients in the training set. The ESTIMATE algorithm was employed to evaluate stromal score, immune score, ESTIMATE score and tumor purity score of each patient [26], and the CIBERSORT algorithm was used to estimate the infiltration of 22 immune cell populations. The R “RColorBrewer”, “ggplot2” and “reshape2” packages displayed the infiltration of immune cells in each sample of the training set and the difference in the proportion of immune cells between the high-risk group and the low-risk group. The TIDE algorithm [27] was used to calculate immune dysfunction score, immune exclusion score and immune-suppressive cells (myeloid-derived suppressor cells, MDSCs) in each sample [28] (TIDE: http://tide.dfci.harvard.edu/), thereby predicting the efficacy of immunotherapy in the high-risk group and the low-risk group. The same analysis was verified in the Xiangya cohort.

### Construction and valuation of nomogram

Univariate and multivariate Cox regression analyses were conducted to identify clinically significant factors associated with survival (*P* < 0.05). Based on these prognostic factors, a nomogram [29] was constructed using the nomogram function from the “rms” package. By assigning values to these variables in the nomogram model, the 1-year, 3-year, and 5-year survival outcomes for patients were calculated. The calibrate function was then used to plot calibration curves, assessing the accuracy of the model.

### Cell culture and patient sample collection

The normal human hepatocyte cell line L02 and six human HCC cell lines (97H, LM3, Hep3B, PLC, HepG2, and Huh7) were purchased from the Chinese Academy of Sciences Cell Line Bank. Cells were maintained under standard culture conditions (37 °C, 5% CO₂) using the following media formulations: L02 was cultured in 1640 medium (Gibco, USA) supplemented with 20% fetal bovine serum (Biological Industries, Israel), PLC was cultured in MEM medium (Gibco, USA) supplemented with 10% fetal bovine serum (FBS, Biological Industries, Israel), and the remaining cells were cultured in DMEM medium (Gibco, USA) containing 10% fetal bovine serum (FBS, Biological Industries, Israel). For clinical samples, paired tumor and adjacent non-tumor tissues (*n* = 60) were obtained from HCC patients undergoing surgical resection at Xiangya Hospital, Central South University. All samples were immediately transferred to − 80 °C via liquid nitrogen after resection. This study was approved by the Ethics Committee of Xiangya Hospital of Central South University.

### Western bloting assays

The collected samples were placed in RIPA buffer (Beyotime, China) containing 1:100 protease and phosphatase inhibitors (Beyotime, China) to extract proteins, and tissue samples were added with protein grinding beads. After the samples were transferred to ice and ultrasonically lysed, they were centrifuged at 12,000 rpm for 30 min at 4℃. The supernatant was aspirated and added with 25x loading buffer (Beyotime, China) to dilute the protein sample, and heated at 95–100℃ for 10 min to denature. The proteins separated by SDS-polyacrylamide gel electrophoresis (SDS-PAGE) were transferred to a 0.45 μm PVDF membrane (Millipore, USA), and then incubated with 5% skim milk at room temperature for 90 min, and the primary antibody G6PD (ab133525, Abcam, UK) diluted 1:1000 was added and incubated at 4 °C overnight. Following three 10-min washes with TBST at room temperature, the membranes were incubated with horseradish peroxidase (HRP)-conjugated goat anti-rabbit secondary antibody (SA00001-2, Proteintech, China) at 1:5000 for 1 h at room temperature, and the signals of protein expression were visualized using the Amersham™ ImageQuant 800 system (Cytiva, USA).

### Immunohistochemistry (IHC)

Fresh HCC tissues were fixed with 4% formalin, dehydrated, paraffin-embedded and prepared into tissue microarrays. After xylene dewaxing and hydration, the tissue microarrays were microwave-heated for antigen retrieval in 1xEDTA antigen retrieval solution (AWI0117a, Abiowell, China). After cooling to room temperature, the endogenous peroxidase of the tissue microarray was blocked with 3% H2O2 at room temperature for 15 min, followed by blocking with 3% BSA diluted in PBS solution for 30 min to reduce nonspecific binding sites. The tissue microarray was incubated in the primary antibody G6PD diluted 1:200 at 4 °C overnight. After incubation with biotin-labeled IgG (ZSGB-BIO, PV-9000) at room temperature for 30 min, the tissue microarrays were stained with DAB (ZSGB-BIO, ZLI-9018) and hematoxylin, permeabilized with xylene and graded alcohol, and then mounted with neutral gum. Each tissue was scored for staining intensity and the percentage of positive cells. Staining intensity was graded as: 0 (negative), 1 (weak positive, light yellow), 2 (positive, brown), or 3 (strong positive, dark brown). The proportion of positive cells was categorized as: 1 (< 25%), 2 (26–50%), 3 (51–75%), or 4 (> 75%). A final immunoreactivity score was calculated by multiplying the intensity score by the percentage score.

### Multiplex immunohistochemistry

To assess the differential expression of regulatory T cells (Tregs) in the tumor microenvironment between high-risk and low-risk patient groups, we performed multiplex tyramide signal amplification (TSA) immunofluorescence staining using a fluorescent immunohistochemistry kit (AWI0693, Abiowell, china). Paraffin sections were dewaxed, hydrated, microwaved for antigen retrieval, blocked with 3% hydrogen peroxide solution, and blocked with BSA in sequence, and the steps and experimental conditions were similar to the IHC method described above. Firstly, the sections were incubated with the GCP3 primary antibody (A21976, Abcam, UK) at 4 °C overnight. Secondly, the sections were incubated with HRP Ms & Rb at room temperature for 30 min, and then incubated with 520 fluorescent dye at room temperature for 10 min. Sections were sequentially incubated with CD4 (1:1000, ab133616, Abcam, UK) and FOXP3 (1:200 dilution, ab215206, Abcam, UK) overnight at 4 °C and incubated with 690 and 570 fluorescent dyes for 10 min at room temperature, respectively. Antigen retrieval was performed with EDTA buffer between rounds of tyramide signal amplification to prevent cross-reactivity. Finally, slides were counterstained with DAPI for 10 min. Images were acquired using a multispectral pathology imaging system.

### Single cell RNA-seq analysis

Data normalization was performed using Seurat (v.4.2.0), and cells expressing less than 300 molecular identifiers and more than 20% of mitochondrial reads were excluded. The top 3000 variable genes were further clustered, and the first 28 principal components were further dimensionalized using UMAP. Cell populations were manually annotated according to previous literature [30]. The ssGSEA algorithm was used to calculate the tumor cell risk score of the scRNA-seq dataset.

### Statistical analysis

R (version 4.3.2) and GraphPad Prism 9 software were used for statistical analysis. The t-test was applied for differential expression analysis of risk scores across different clusters, as well as stromal scores, immune scores, and tumor purity between different risk groups. Survival analysis between the high-risk and low-risk groups was performed using the log-rank test, while the chi-square test was used to evaluate immune therapy responses. The Spearman method was employed to analyze the correlation between model genes and immune cells. P value < 0.05 was defined as statistically significant.

## Results

### Identification of DEGs related to de novo lipogenesis and functional enrichment analysis in HCC

**Fig. 1:**
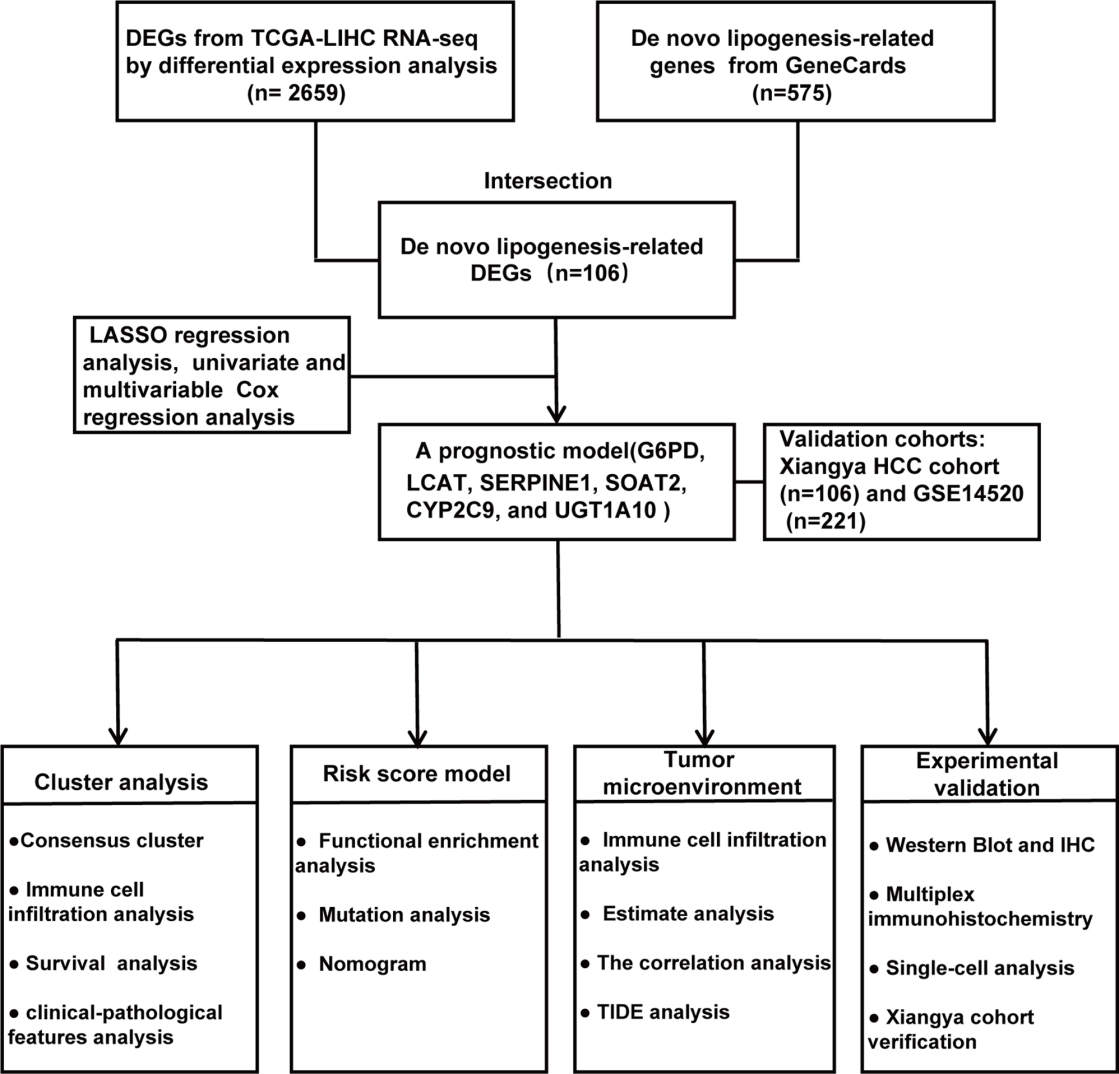
Flowchart of this Study

**Fig. 2:**
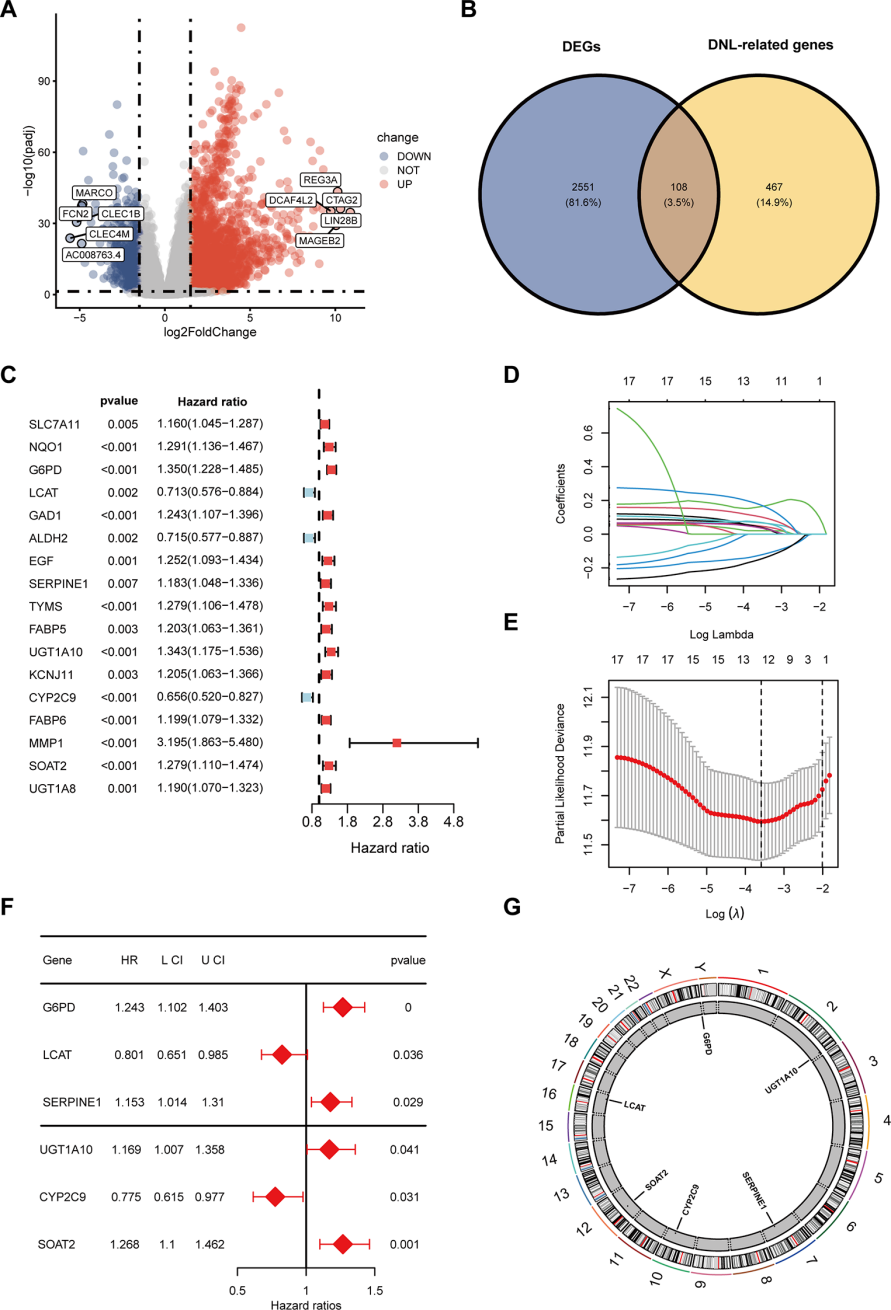
Screening DEGs of De novo Lipid Synthesis and Constructing a Prognostic Risk Model in the TCGA-LIHC Cohort. (**A**)The volcano plot showed DEGs between HCC tumor and normal groups, highlighting the|log2FoldChange| top 10 genes. (**B**) The Venn diagram shows that 108 overlapping genes were identified from the intersection of 2,659 DEGs and 574 DNL-related genes. (**C**) Univariate Cox regression analysis showed that 17 genes were associated with the prognosis of HCC patients. (**D**) LASSO regression of the 12 OS-related genes. The coefficient path plot showed the trajectories of the regression coefficients for each curve as the regularization parameter λ changes. The cross-validation plot shows the cross-validation error of the model at different λ values, with the horizontal axis representing the regularization parameter λ and the vertical axis representing partial likelihood deviance. (**E**) Multivariate Cox regression analysis showed that six genes were associated with the prognosis of HCC patients. (**F**) The circular chromosome plot visualizes the chromosomal locations of the model genes

A simplified schematic workflow illustrating the experimental procedures was presented in Fig. 1. 2659 DEGs including 546 downregulated genes and 2113 upregulated genes (Additional file 1: Table S2), were screened and visualized via volcano maps between the tumor and adjacent tissue samples in the TCGA-LIHC cohort (Fig. 2A). The expression patterns of the top 20 most significantly upregulated and downregulated genes were illustrated in a heatmap (Additional file 2: Fig. S1A). In addition, the volcano plot also showed the differentially expressed genes between tumor tissue and adjacent tissues in the Xiangya HCC cohort (Additional file 2: Fig. S1B). Subsequent integrative analysis of 575 de novo lipogenesis-related genes curated from GeneCards (Additional file 1: Table S2) with our 2,659 DEGs yielded 108 candidate de novo lipogenesis-related DEGs potentially involved in HCC pathogenesis (Additional file 1: Table S2).

**Fig. 3:**
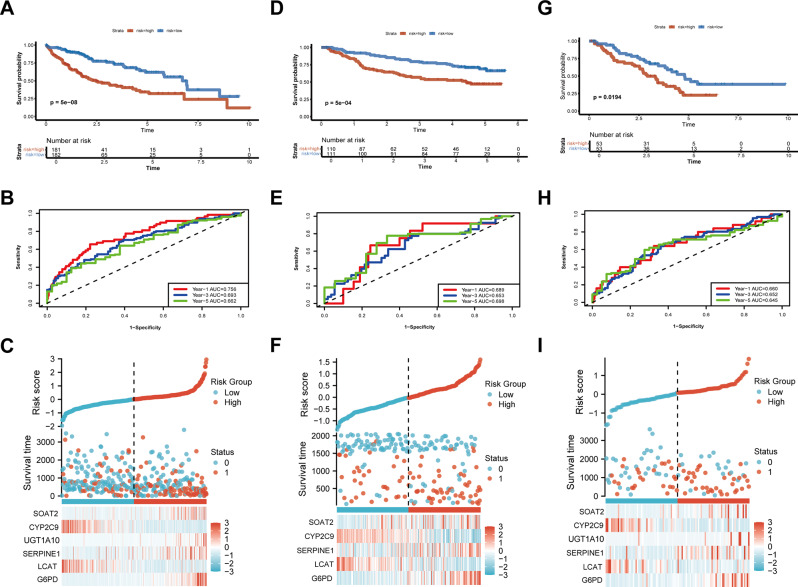
The performance of the prognostic risk model was evaluated using the TCGA-LIHC cohort and external validation cohorts. (**A**)Kaplan–Meier curves of the OS of patients in the TCGA-LIHC training cohort. (**B**) ROC curves for predicting 1-, 3-, and 5-year OS in the TCGA-LIHC training cohort. (**C**) The distribution of risk score, survival status (1 indicate dead,0 indicate alive) and the gene expression of 6 model genes in the high- and low-risk groups in the TCGA-LIHC training cohort. (**D**) Kaplan–Meier curves of the OS of patients in the GSE14520 cohort. (**E**) ROC curves for predicting 1-, 3-, and 5-year OS in the GSE14520 cohort. (**F**) The distribution of risk score, survival status (1 indicate dead,0 indicate alive) and the gene expression of 6 model genes in the high- and low-risk groups in the GSE14520 cohort. (**G**) Kaplan–Meier curves of the OS of patients in the Xiangya HCC cohort. (**H**) ROC curves for predicting 1-, 3-, and 5-year OS in the Xiangya HCC cohort. (**I**) The distribution of risk score, survival status (1 indicate dead,0 indicate alive) and the gene expression of 6 model genes in the high- and low-risk groups in the Xiangya HCC cohort

### Construction and validation of a de novo lipogenesis‑related risk signature

Univariate Cox regression analysis further identified 17 out of 108 de novo lipogenesis-related DEGs as significantly associated with HCC prognosis (*P* < 0.01, Fig. 1C). subsequent LASSO regression analysis refined this set to 12 genes, with multivariate Cox regression further reducing the number to 6 prognostic biomarkers (Fig. 1D and E). We ultimately established a prognostic model incorporating six de novo lipogenesis-related DEGs: G6PD, LCAT, SERPINE1, SOAT2, CYP2C9, and UGT1A10. Notably, while traditional biomarkers such as AFP, GPC3, and TP53 [31] primarily influence tumor growth, differentiation, proliferation, and genomic stability, our model genes exhibit distinct functional relevance to lipid metabolism pathways in HCC. This distinction underscores the complementary value of our model, highlighting its unique predictive significance for HCC. The K-M curves indicated that patients with high expression of G6PD (*P* = 0.0029) and SERPINE1 (*P* = 0.02) had poorer prognosis compared to those with low expression (Additional file 2: Fig. S1C-S1D). However, patients with higher expression levels of LCAT (*P* = 0.00035) and CYP2C9 (*P* = 0.0019) had better prognosis than those with low expression (Additional file 2: Fig. S1E-S1F). The expression levels of UGT1A10 (*P* = 0.07) and SOAT2 (*P* = 0.83) had no impact on prognosis (Additional file 2: Fig. S1G-S1H).

Patients were divided into high-risk and low-risk groups based on the median risk score. The high-risk group demonstrated significantly shorter overall survival (OS) in Kaplan-Meier analysis (Fig. 2A). The predictive accuracy of the prognostic risk model was evaluated using the ROC curve, with AUC values of 0.756, 0.693, and 0.662 for 1, 3, and 5 years, respectively (Fig. 2B). A comprehensive heatmap visualized the interrelationships between risk scores, survival outcomes, and model gene expression patterns (Fig. 2C). These results suggested the robustness of our risk model in predicting prognosis for HCC patients. External validation using the GSE14520 dataset, Xiangya HCC cohort, and ICGC-LIRI cohort consistently replicated these findings. High-risk patients exhibited worse prognosis across all validation sets (Fig. 2D and G; Additional file 2: Fig. S2A-S2B). The model maintained robust predictive performance, with 1-, 3-, and 5-year AUC values of 0.689, 0.653, and 0.698 in GSE14520 (Fig. 3E); 0.660, 0.652, and 0.645 in the Xiangya cohort (Fig. 3H); and 0.788 and 0.736 for 1- and 3-year predictions in ICGC-LIRI (Additional file 2: Fig. S2B). Risk factor heatmaps consistently demonstrated inverse correlations between risk scores and survival duration (Fig. 3F and I).

**Fig. 4:**
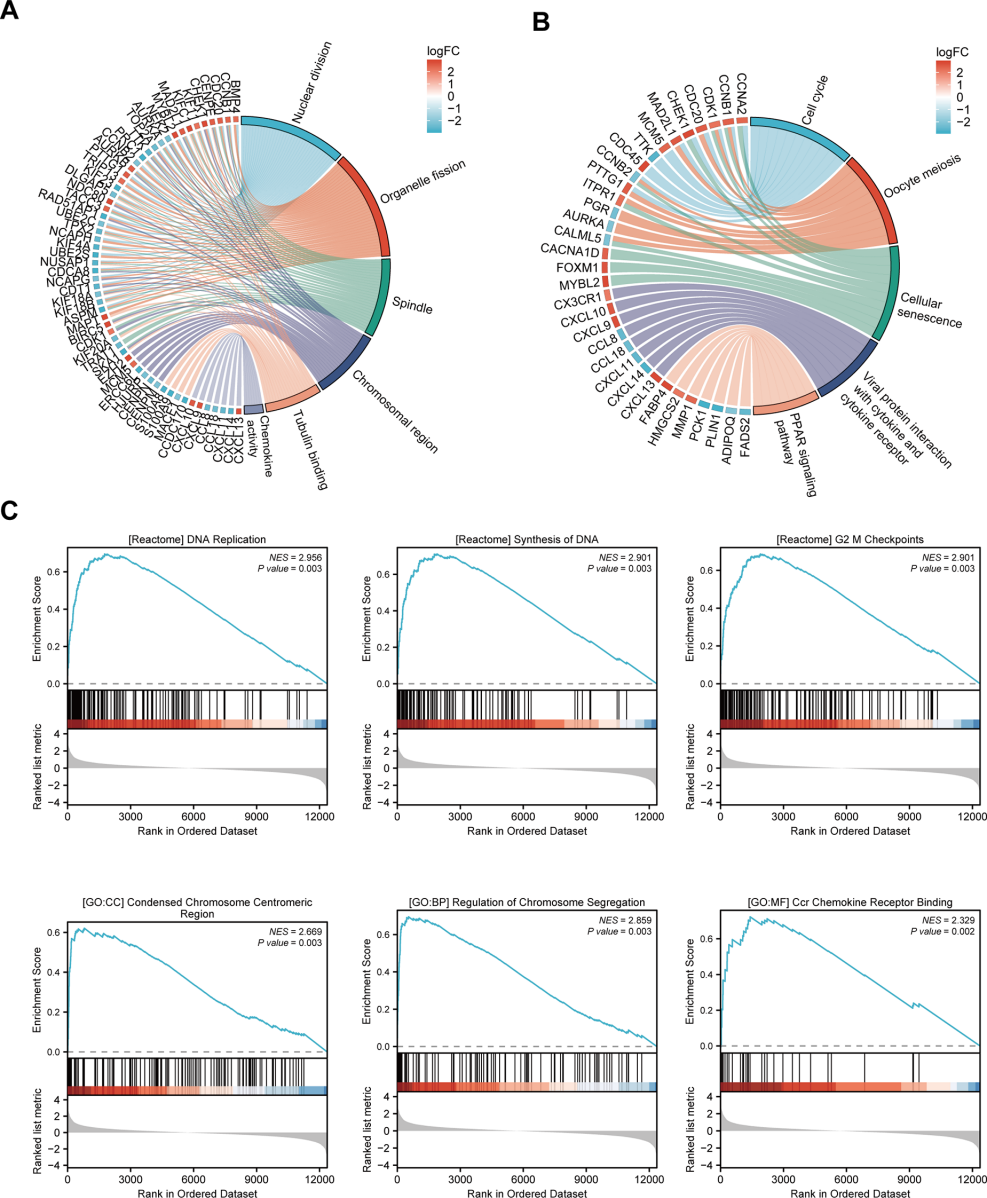
Enrichment analysis of high- and low-risk groups in the TCGA-LIHC cohort. **(A**) GO enrichment chord diagram, with the left semicircle representing enriched genes and the right semicircle showing six enriched GO pathways in different colors. (**B**) KEGG enrichment chord diagram, where the left semicircle represented enriched genes and the right semicircle displayed five enriched KEGG pathways in different colors. (**C**) GSEA analysis showed gene sets enriched in the high-risk group

### Functional enrichment analysis of the DEGs in high‑risk and low‑risk groups

We further performed functional enrichment analysis on the 2,280 DEGs between the high-risk and low-risk groups (Additional File 1: Table S4). GO enrichment analysis indicated that the differential genes were involved in biological processes (BP) such as nuclear division and organelle fission. In cellular components (CC), they were mainly enriched in spindle and chromosomal region. The main molecular functions (MF) included microtubule binding and chemokine activity (Fig. 3A, Additional File 1: Table S5). KEGG pathway analysis identified the top five significantly enriched pathways as: cell cycle, oocyte meiosis, cellular senescence, viral protein interaction with cytokine and cytokine receptor and PPAR signaling pathway (Fig. 3B, Additional File 1: Table S6). Gene Set Enrichment Analysis (GSEA) demonstrated significant associations between the risk score and several critical pathways in the high-risk group, including DNA replication, G2 M checkpoints, synthesis of DNA, regulation of chromosome segregation, condensed chromosome centromeric region and CCR chemokine receptor binding in the high-risk group (Fig. 3C).

### Cluster analysis of de novo lipogenesis‑related genes

The TCGA-LIHC cohort samples were divided into two clusters (Fig. 4A and C). Dimension reduction visualization using UMAP and t-SNE algorithms revealed clear separation between the two clusters with minimal overlap, while demonstrating strong intra-cluster homogeneity (Fig. 4D and F). Subsequent immune infiltration analysis identified significant differential expression patterns across 9 immune cell subtypes between the clusters (Fig. 4E). Survival analysis indicated that Cluster I exhibited significantly poorer OS compared to Cluster II (Fig. 4F). A comprehensive heatmap displayed the distribution of clinicopathological features and expression of the 6 model genes across both clusters (Fig. 4H).

**Fig. 5:**
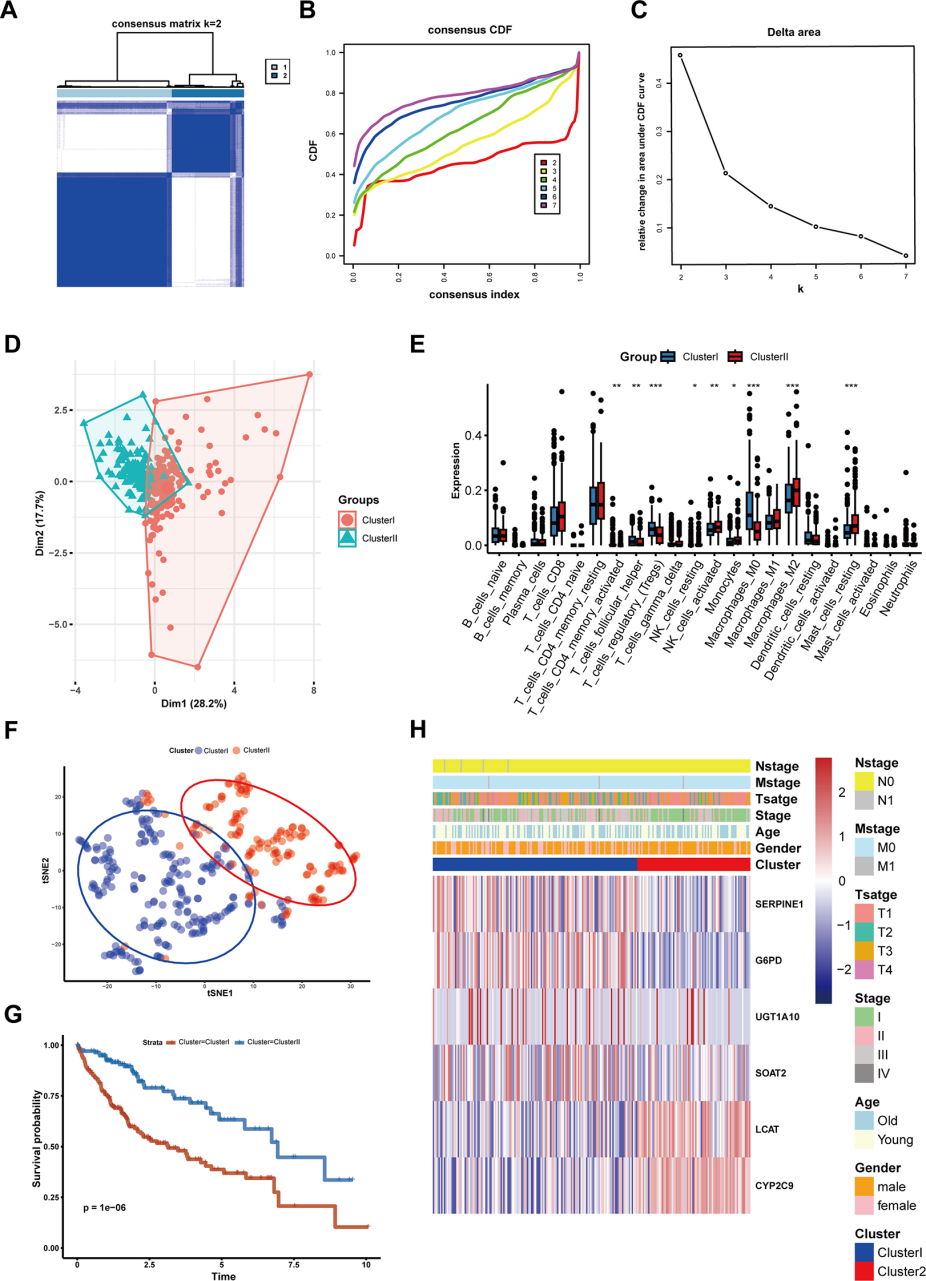
Different de novo lipid synthesis patterns were identified in 365 HCC patients from the TCGA-LIHC cohort. (**A**) Heatmap of the consensus matrix for two clusters (k = 2). (**B**) The CDF plot of the consensus matrix for k = 2–7. (**C**) The Delta area plot indicated that k = 2 was the optimal number of clusters. (**D**) The Dim plot showed the spatial distribution of the two clusters. (**E**) Immune cell infiltration differences between the two clusters. (**F**) t-SNE analysis of the two clusters. (**G**) Survival analysis of de novo lipid synthesis clusters based on OS (log-rank test). (**H**) Complex heatmap showing expression levels of model genes and the distribution of clinical characteristics in de novo lipid synthesis clusters. * P < 0.05, ** P < 0.01, *** P < 0.001

There were significant differences in tumor stage and T stage between the clusters, with no noticeable differences in gender or age (Fig. 5A). The Sankey diagram showed that most samples from cluster I were classified into the high-risk score group, while nearly all samples from cluster II were classified into the low-risk score group. Most deceased patients were in the high-risk score group, whereas the vast majority of cases in the low-risk score group were surviving patients (Fig. 5B). Figure 5C further illustrated that the risk scores in cluster I were significantly higher than those in cluster II. Mutation analysis revealed that the single-gene mutation frequency in the high-risk score group was higher than in the low-risk score group (Fig. 5D and E), with no significant differences in mutation type, Ti/Tv ratio, and tumor mutation burden (TMB) (Additional file 2: Fig. S2C-2G).

**Fig. 6:**
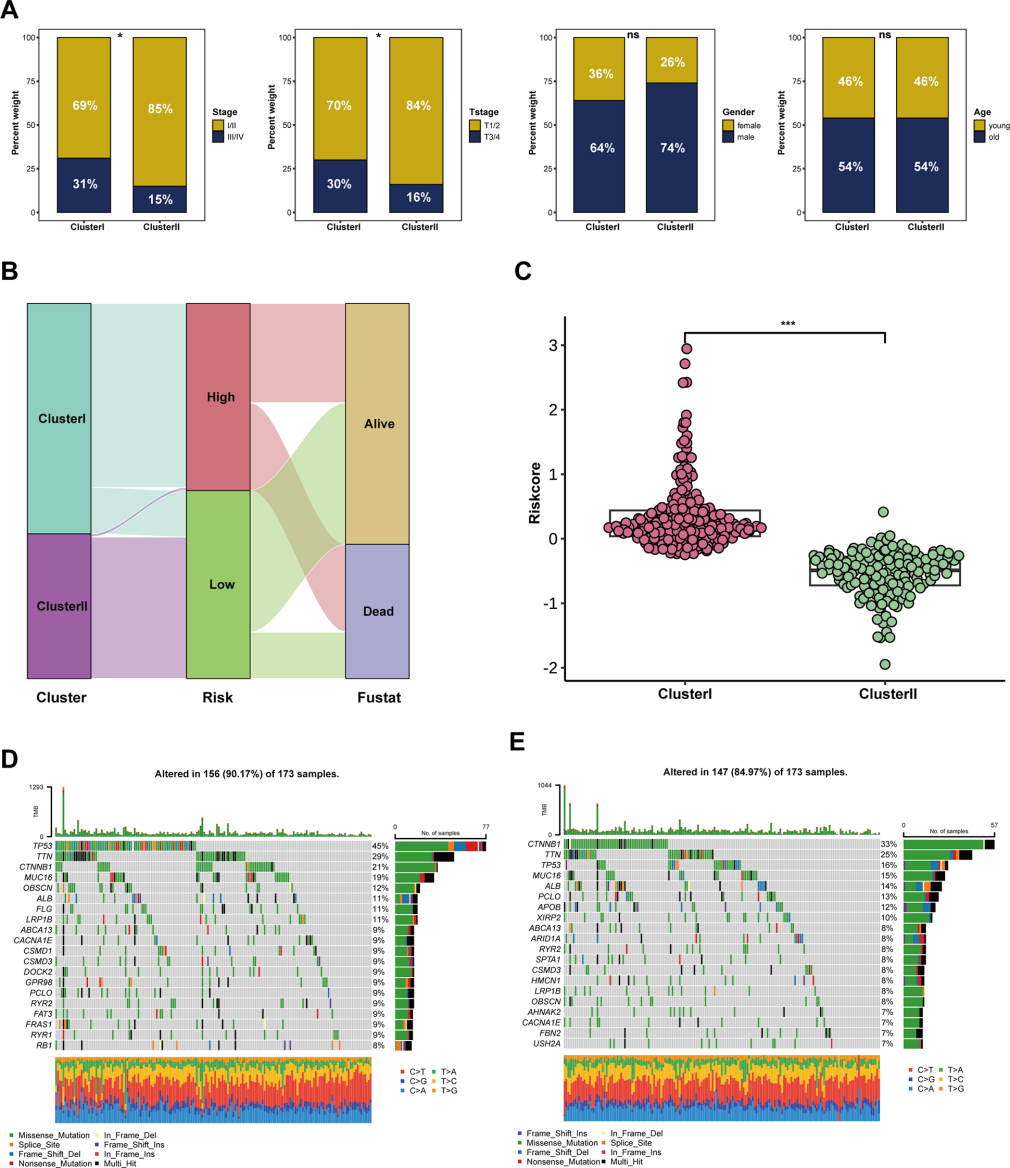
The relationship between clusters and risk scores, and mutation status in the high- and the low-risk groups in TCGA-LIHC cohort. (**A**) Comparison of clinical characteristics between patients in the two clusters. (**B**) Sankey diagram of clusters, risk, and patient survival outcomes. (**C**) Comparison of risk scores between the two clusters. (**D**) The waterfall plot showed the top 20 genes in the high- risk group based on mutation frequency. (**E**) The waterfall plot showed the top 20 genes low-risk group based on mutation frequency. * P < 0.05, *** P < 0.001

### De novo lipogenesis‑related risk score was associated with immune signatures and immunotherapy responses in HCC

The tumor immune microenvironment (TIME) was closely associated with treatment efficacy and prognosis in patients with malignancies. Consequently, investigating the association between risk scores and immune cell infiltration patterns was of paramount importance. Our analysis revealed that the TIME in HCC patients was predominantly characterized by M2 macrophage infiltration (Additional file 2: Fig. S3A). Activated CD4^+^T cells, Tregs, follicular helper T cells (Tfh), M0 macrophages, and neutrophils were significantly elevated in the high-risk group. Conversely, the low-risk group was primarily characterized by infiltration of NK cells, monocytes, and mast cells, and similar findings were observed in the Xiangya HCC cohort (Fig. 6A and B). Correlational studies of immune infiltration characteristics demonstrated significant positive associations between the risk score and both M0 macrophages and Tregs, while showing negative correlations with activated NK cells and mast cells (Fig. 6C). Similar results were obtained in the Xiangya HCC cohort (Additional file 2: Fig. S3B). However, neither the TCGA-LIHC nor the Xiangya HCC cohorts exhibited significant correlations between risk scores and immune/stromal scores (Additional file 2: Fig. S3C-3D).Further investigation of immunosuppressive cytokine profiles revealed significantly elevated expression of TGF-β and IL-10 in high-risk patients within the TCGA-LIHC cohort (Additional file 2: Fig. S3E-3 F), suggesting that enhanced de novo lipogenesis in these patients may upregulate these cytokines, potentially facilitating Treg recruitment and fostering an immunosuppressive microenvironment.

**Fig. 7:**
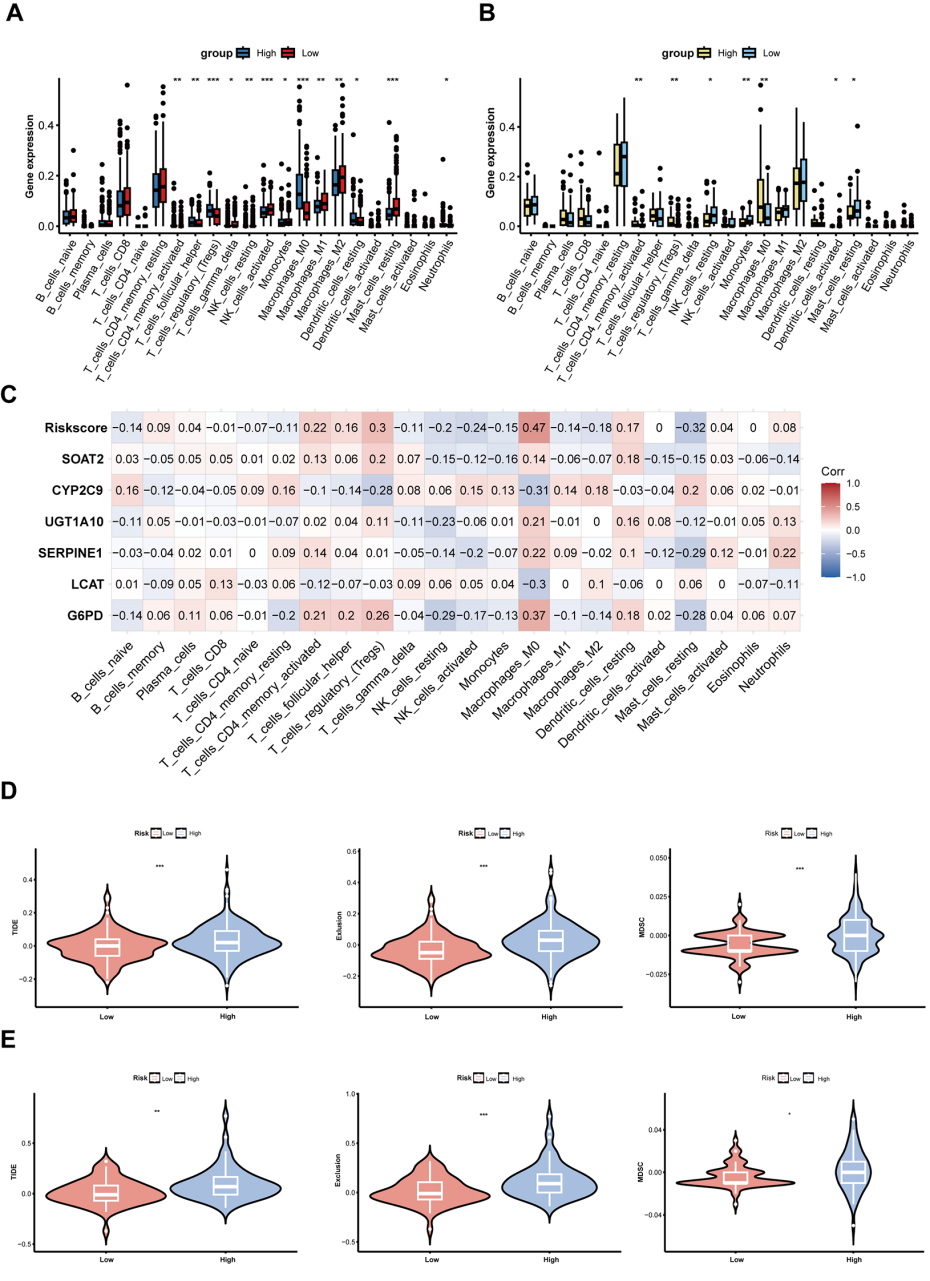
Immune cell infiltration and immune response in the TCGA-LIHC and Xiangya HCC cohorts. (**A**) Comparison of immune cell infiltration between high- and low-risk groups in the TCGA-LIHC cohort. (**B**) Comparison of immune cell infiltration between high- and low-risk groups in the Xiangya HCC cohort. (**C**) Correlation analysis of the six model genes with each immune cell population in the TCGA-LIHC cohort. (**D**) Comparison of TIDE scores, exclusion scores, and MDSCs level between high- and low-risk groups in the TCGA-LIHC cohort. (**E**) Comparison of TIDE scores, exclusion scores, and MDSCs level between high- and low-risk groups in the Xiangya HCC cohort. * P < 0.05, ** P < 0.01, *** P < 0.001

Comprehensive analysis of immunotherapy responsiveness demonstrated that high-risk patients in both cohorts exhibited elevated TIDE and Exclusion scores, along with increased myeloid-derived suppressor cell (MDSC) infiltration (Fig. 6D and E), while patients responding to immunotherapy had lower risk scores (Additional file 2: Fig. S3G-3 H). Although there was no significant difference in dysfunction score between the two groups (Additional file 2: Fig. S3I-3 J), these collective findings strongly implicate immunosuppressive TIME characteristics and immunotherapy resistance as potential determinants of poor prognosis in high-risk HCC patients.

### Stablishment of nomogram for accurate patient prognosis prediction

**Fig. 8:**
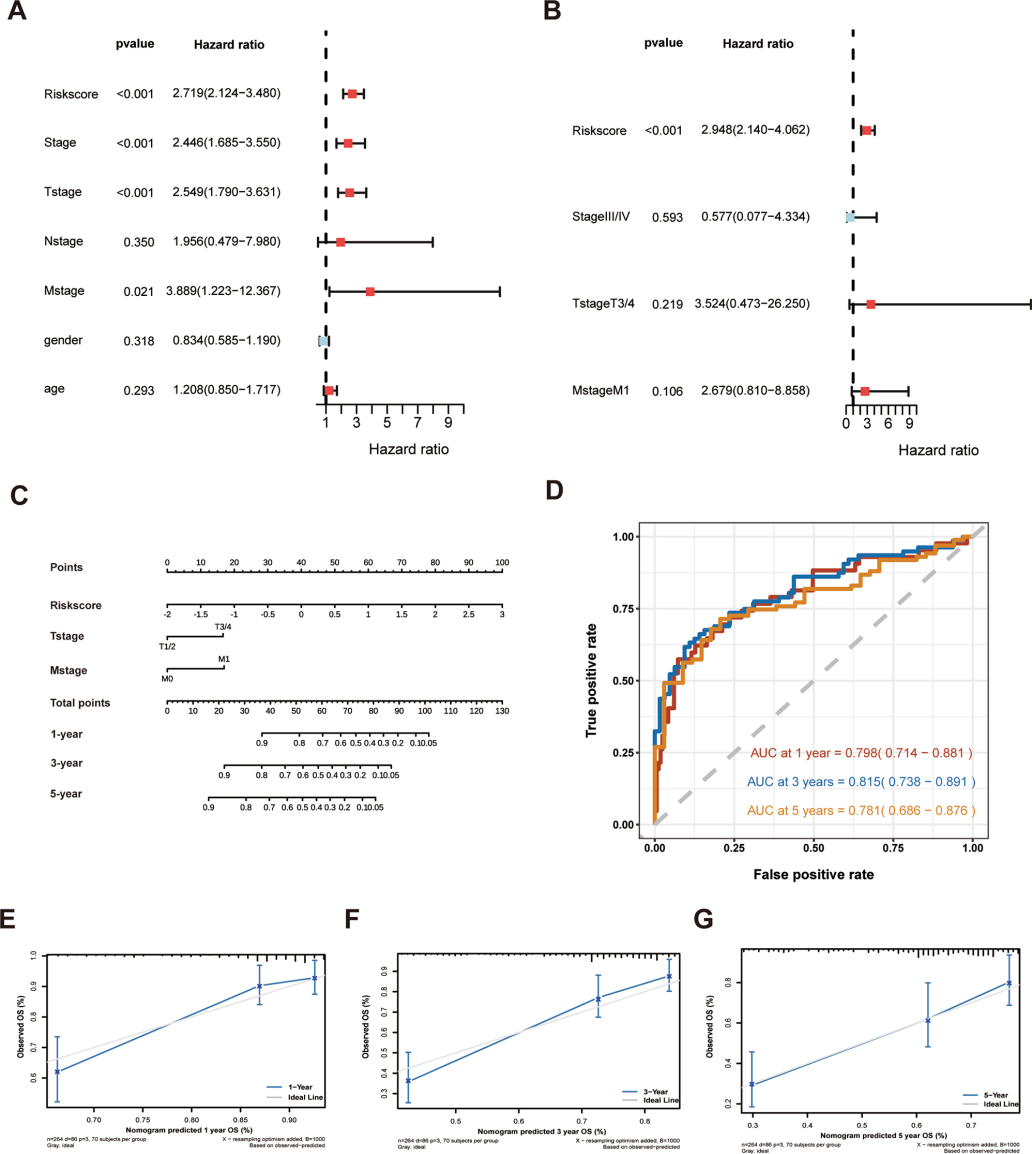
The nomogram integrated multiple survival-influencing factors to provide personalized prognosis assessment for patients. (**A**) Univariate Cox regression analysis identified factors associated with patient survival. (**B**) Multivariate Cox regression analysis identified independent prognostic factors associated with patient survival. (**C**) The nomogram plot. (**D**) The area under the curve (AUC) values for 1 year, 2 years, and 3 years. (**E**) Nomogram prediction of 1-year survival probability. (**F**) Nomogram prediction of 3-year survival probability. (**G**) Nomogram prediction of 5-year survival probability

Firstly, we conducted univariate Cox regression analysis to preliminarily screen factors affecting patient prognosis, the results indicated that risk score, tumor stage, T stage, and M stage significantly impacted patient prognosis (Fig. 8A). Patients with tumor stage III/IV had higher risk scores compared to those with stage I/II, and patients with T3/4 had higher risk scores compared to those with T1/2 (Additional file 2: Fig. S4A-4B), findings that were subsequently validated in the Xiangya HCC cohort (Additional file 2: Fig. S4E-4 F). However, there was no difference in risk scores between N0 and N + or between M0 and M1 groups (Additional file 2: Fig. S4C-4D). Multivariate Cox regression analysis further confirmed that the risk score served as an independent prognostic factor (Fig. 8B). In the Xiangya HCC cohort, significant differences were observed between high-risk and low-risk groups regarding survival status, tumor stage, and T stage (Table 1). We subsequently constructed a nomogram to quantitatively predict patient prognosis and facilitate clinical decision-making. The model demonstrated good predictive performance, with a C-index of 0.737, indicating good prediction performance (Fig. 8C). Comparative analysis revealed that our DNL-related prognostic model outperformed existing HCC prognostic models (including BCLC and CNLC), achieving superior AUC values of 0.798, 0.815, and 0.781 for 1-year, 3-year, and 5-year survival predictions, respectively. These values were significantly higher than those of conventional models (typically ranging from 0.55 to 0.75; Fig. 6D). Furthermore, the nomogram significantly improved the accuracy of 1-, 3-, and 5-year survival predictions (Fig. 8E-G).

**Table 1:** Clinicopathological characteristics and of high-risk and low-risk group in the Xiangya HCC cohort

Characteristics	High-risk	Low-risk	*P* value
n	53	53	
Fustat, n (%)			0.050
1	35 (33%)	25 (23.6%)	
0	18 (17%)	28 (26.4%)	
Tstage, n (%)			0.004
T1/2	7 (6.7%)	20 (19%)	
T3/4	45 (42.9%)	33 (31.4%)	
Nstage, n (%)			0.976
N0	47 (44.8%)	49 (46.7%)	
N+	5 (4.8%)	4 (3.8%)	
Mstage, n (%)			0.692
M0	50 (47.6%)	49 (46.7%)	
M1	2 (1.9%)	4 (3.8%)	
Stage, n (%)			0.004
I/II	7 (6.6%)	20 (18.9%)	
III/IV	46 (43.4%)	33 (31.1%)	
Gender, n (%)			0.791
F	8 (7.5%)	9 (8.5%)	
M	45 (42.5%)	44 (41.5%)	
Age, n (%)			1.000
Young	38 (35.8%)	38 (35.8%)	
Old	15 (14.2%)	15 (14.2%)	
AFP, n (%)			0.059
1	41 (38.7%)	32 (30.2%)	
0	12 (11.3%)	21 (19.8%)	
Cirrhosis, n (%)			0.652
1	14 (13.2%)	12 (11.3%)	
0	39 (36.8%)	41 (38.7%)	

### Validation of DNL-related genes expression in the real-world cohort

**Fig. 9:**
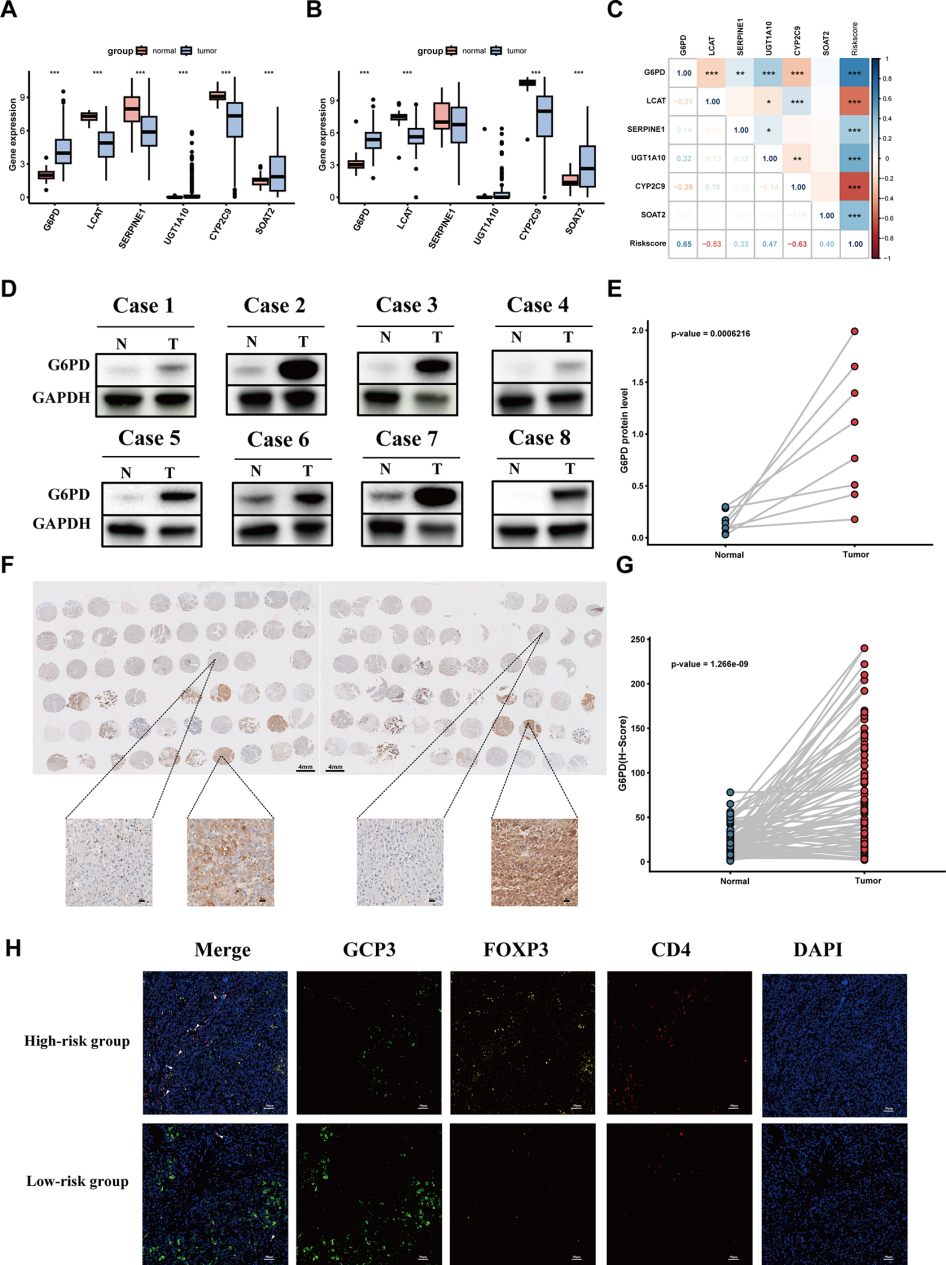
Validation of model gene expression levels and Treg cell infiltration. (**A**) mRNA expression levels of model genes in tumor and adjacent normal tissues in the TCGA-LIHC cohort. (**B**) mRNA expression levels of model genes in tumor and adjacent normal tissues in the Xiangya HCC cohort. (**C**) Correlation analysis between model genes and risk scores. (**D**) Protein expression levels of G6PD in 8 pairs of HCC and paired adjacent tissues. (**E**) Protein expression statistical chart. (**F**) Immunohistochemistry chips showed G6PD expression in 85 pairs of HCC and paired adjacent tissues. (**G**) G6PD expression statistical chart. (**H**) Representative multiplex immunofluorescence images of Treg cells in high- and low-risk groups. *** P < 0.001

Comparison of the expression levels of six model genes (G6PD, LCAT, SERPINE1, SOAT2, CYP2C9, UGT1A10) in 369 HCC tissues and 50 adjacent HCC tissues from the TCGA-LIHC cohort showed that G6PD, UGT1A10 and SOAT2 were highly expressed in HCC tissues and lowly expressed in the adjacent tissues, while LCAT, SERPINE1 and CYP2C9 were lowly expressed in HCC tissues and highly expressed in the adjacent tissues. We obtained similar results in the Xiangya cohort, except that there was no difference in the expression of SERPINE1 and UGT1A10 (Fig. 9A and B). Correlation analysis indicated that G6PD, SERPINE1, SOAT2 and UGT1A10 were highly positively correlated with the risk score, whereas LCAT and CYP2C9 were negatively correlated with the risk score. Notably, G6PD emerged as the most significant determinant of risk scores (Fig. 9C). To quantitatively assess each gene’s contribution to model performance, we employed SHAP analysis. This revealed G6PD as the most influential feature in the predictive model (Additional file 2: Fig. S5A). The results revealed that G6PD, as the most important gene, was positioned at the top (Additional file 2: Fig. S5A). Subsequent validation experiments using tissue microarrays and Western blotting confirmed significant overexpression of G6PD protein in HCC tissues compared to matched paracancerous controls (Fig. 9D and F, Additional file 2: Fig. S5B). In vitro studies further demonstrated elevated G6PD expression in multiple HCC cell lines (97 H, LM3 and PLC; Additional file 2: Fig. S5C).

**Fig. 10:**
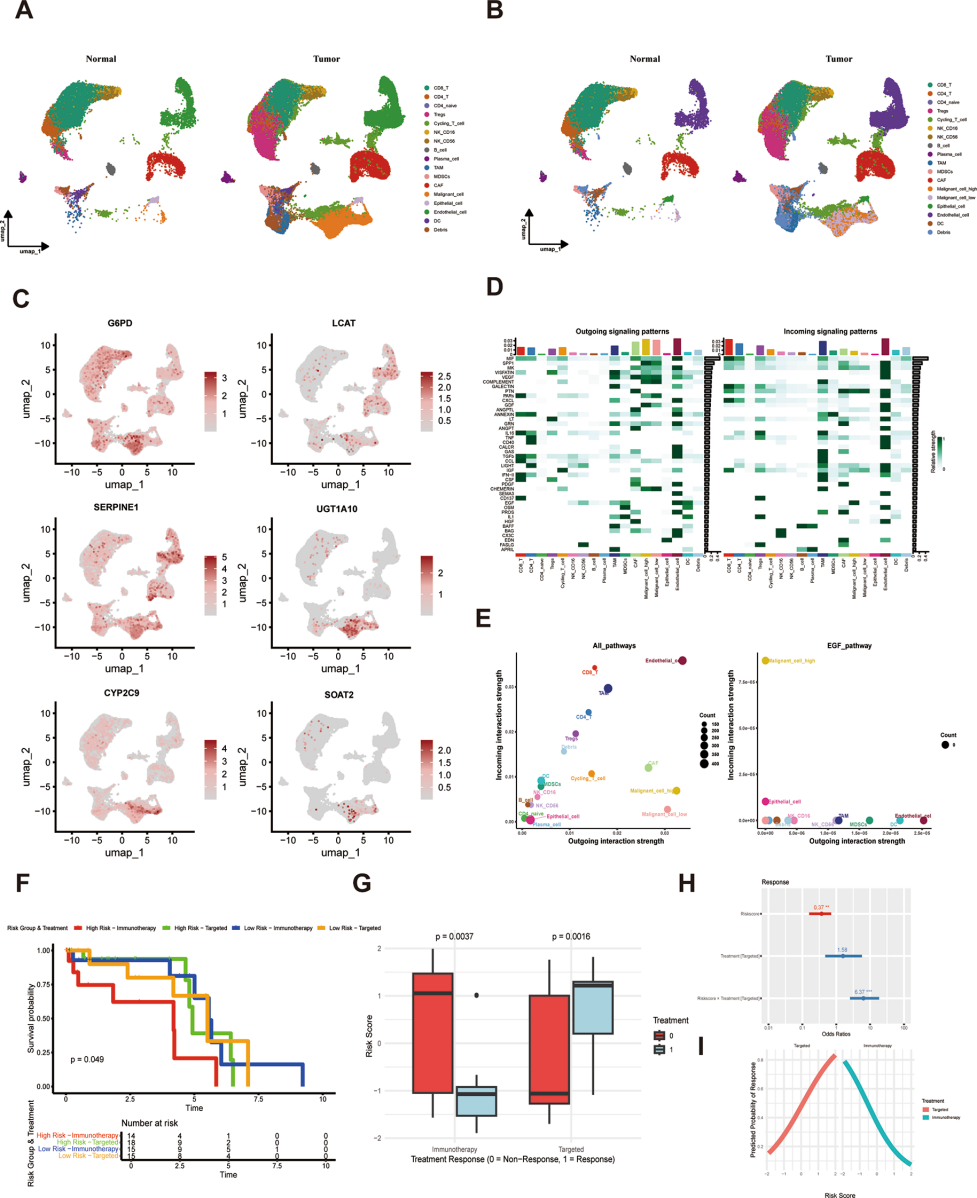
Single-cell level analysis of model genes. **(A**) UMAP plot showed the distribution of 17 cell subpopulations in tumor and normal tissues. (**B**) Distribution of tumor cell subpopulations with high and low risk scores in the UMAP plot. (**C**) Expression levels of model genes across the 17 cell subpopulations. (**D**-**E**) Enrichment analysis of incoming and outgoing signaling patterns in high- and low-risk tumor cell subpopulations. (**F**) The Kaplan-Meier curves for patients in the high-risk targeted therapy group, low-risk targeted therapy group, high-risk immunotherapy group, and low-risk immunotherapy group were analyzed. (**G**) The box plot illustrated the treatment response of high and low-risk score HCC patients across different treatment groups. (**H**) The forest plot illustrated the relationship between risk scores and response to targeted therapy. (**I**) The interaction plot illustrated the relationship between risk scores and treatment response by treatment type, with the X-axis representing risk scores and the Y-axis indicating the probability of treatment response

Multiplex immunohistochemical staining (GPC3, CD4, Foxp3) revealed distinct tumor immune microenvironments between risk groups. The high-risk group exhibited significantly greater infiltration of regulatory T cells (Tregs, CD4 + Foxp3+), indicative of an immunosuppressive tumor microenvironment (Fig. 9H). Single-cell RNA sequencing analysis delineated the spatial expression patterns of model genes across cell subpopulations (Fig. 10A and C). Communication analysis showed that compared with the low-risk tumor cell group, EGF pathway was significantly enriched in high-risk tumor cell populations (Fig. 10D and E).

Clinical response analysis in the validation cohort showed divergent treatment outcomes: immunotherapy responders had significantly lower risk scores than non-responders, while the opposite was true for targeted therapy (*P* = 0.0016, Fig. 10G). Survival analysis indicated that in the high-risk group, patients had better survival outcomes after receiving targeted therapy compared to immunotherapy, whereas in the low-risk group, patients had better survival outcomes after receiving immunotherapy compared to targeted therapy (Fig. 10F). Logistic regression modeling confirmed these findings, demonstrating significant interaction effects between risk scores and treatment modalities (Fig. 10H and I). These findings demonstrated a significant treatment-specific association between risk stratification and therapeutic efficacy. Specifically, patients stratified into the high-risk group exhibited enhanced responsiveness to targeted therapies, whereas those in the low-risk category showed more favorable outcomes with immunotherapeutic interventions.

## Discussion

HCC poses challenges for prognostic and treatment response predictions due to its highly complex and heterogeneous molecular phenotypes [32–34]. Therefore, effective molecular biomarkers are essential for identifying different HCC subtypes and providing more personalized prognostic assessments and treatment strategies for HCC patients. Tumor cells adapt to their high energy demands by altering lipid metabolism, with enhanced DNL being a major source of lipid utilization [35]. Certain signaling pathways in tumor cells, such as the PI3K/Akt/mTOR pathways, regulate DNL activity [36]. These pathways enhance DNL by modulating the expression of related enzymes, such as FASN and ACC, promoting tumor growth. Inhibiting these enzymes could effectively slow tumor growth and metastasis, making them important pharmacological targets in various cancers [37–41]. Increasing evidence suggests that genes related to de novo lipogenesis can serve as biomarkers for the diagnosis and treatment of malignant tumors.

G6PD, a rate-limiting enzyme in the pentose phosphate pathway (PPP), catalyzes the conversion of glucose-6-phosphate to 6-phosphogluconate while generating NADPH, an essential reducing equivalent for lipid biosynthesis [42]. Multiple studies have shown that the upregulation of G6PD is associated with tumor progression, increased aggressiveness, and drug resistance [43, 44]. G6PD is highly expressed in prostate cancer and is strongly correlated with bone metastasis and poor prognosis in patients [45]. Aldolase B inhibits HCC progression by directly binding to and suppressing G6PD [46]. Inhibition of the PI3K/AKT/mTOR pathway promotes G6PD protein degradation, reducing radiotherapy resistance in small cell lung cancer [47]. Furthermore, patients with low G6PD expression undergoing immune checkpoint inhibitor therapy for lung cancer and melanoma have better prognoses [48]. These studies suggest that G6PD’s central role in tumor metabolism makes it not only a target for cancer therapy but also an ideal target for combination treatments. In our predictive model, G6PD exhibited the strongest correlation with risk scores. Subsequent validation confirmed its significant upregulation in HCC tissues compared with adjacent non-tumorous tissues at both transcriptional and translational levels, which was associated with poor patient prognosis. Consistent with previous reports, we observed elevated G6PD expression in highly invasive HCC cell lines (97 H, LM3). Additionally, SERPINE1, UGT1A10, and SOAT2 showed positive correlations with risk scores, suggesting their roles as unfavorable prognostic factors. Conversely, LCAT and CYP2C9 demonstrated negative correlations with risk scores, indicating their potential protective functions in HCC progression.

SERPINE1 plays a crucial role in regulating the fibrinolytic system, extracellular matrix remodeling, and the tumor microenvironment (TME) [49]. Dan Zhang et al. reported that after anti-cancer treatment, senescent tumor cells (STCs) gradually accumulate in colorectal cancer and promote tumor progression by releasing extracellular vesicles rich in SERPINE1. Consequently, increased SERPINE1 expression after treatment correlates with shorter disease-free survival (DFS) or progression-free survival (PFS), suggesting that SERPINE1 is associated with tumor progression and may be a potential therapeutic target [50]. Additionally, Hong et al. found that CD248 + pericytes express SERPINE1, which was linked to increased tumor size in lung cancer, and high SERPINE1 expression was associated with poor survival in lung cancer patients [51]. This aligned with our findings that SERPINE1 was highly expressed in HCC and was correlated with poor prognosis in patients. Regarding cholesterol metabolism, SOAT2 participates in tumor metabolic reprogramming. However, Carolin et al. reported that SOAT1 exhibited superior prognostic value over SOAT2 in high-risk prostate cancer However, Carolin et al. mentioned that SOAT1 was a better prognostic marker for high-risk prostate cancer compared to SOAT2 [52]. Our study similarly showed that although SOAT2 was highly expressed in HCC, it was not an effective predictor of prognosis for HCC patients, indicating that further research is needed to understand the molecular functions of SOAT2 more comprehensively. LCAT also plays a key role in cholesterol metabolism, promoting the production of high-density lipoprotein cholesterol (HDL-C) through the LDLR and SCARB1 pathways. High levels of HDL-C impair the maturation of SREBP2, ultimately inhibiting cholesterol biosynthesis and suppressing HCC cell proliferation [53]. Long’s research suggested that LCAT was a protective gene and was associated with a favorable prognosis in liver cancer patients [54]. This was consistent with our study, which showed that LCAT was downregulated in HCC, and patients with higher LCAT expression had better prognoses. Studies had shown that CYP2C9 was downregulated in HCC and was positively associated with overall survival in patients [55], and our findings in both the training and validation sets supported this. UGT1A10, a member of the UDP-glucuronosyltransferase (UGT) superfamily, was involved in the metabolism of estrogens and anticancer drugs like tamoxifen [56, 57], suggesting its potential role in cancer development and drug response. In this study, although UGT1A10 was more highly expressed in cancer tissues compared to adjacent normal tissues, its overall expression level was low. Patients with higher UGT1A10 expression had poorer prognoses, but the results were not statistically significant, requiring further validation in larger cohorts.

Functional enrichment analyses (GO, KEGG, and GSEA) revealed significant pathway enrichment differences between high- and low-risk groups, particularly in cell cycle regulation, DNA replication, and chromosome segregation. Cell cycle dysregulation represents a fundamental oncogenic mechanism driving uncontrolled tumor proliferation [58], likely contributing to the adverse prognosis observed in high-risk patients. The TME heterogeneity significantly impacts HCC prognosis and influences immunotherapy responsiveness [59]. Critical TME components including tumor cells, ECM, and immune cells collectively determine tumor aggressiveness and treatment efficacy [60, 61]. Notably, while Tregs facilitate immune evasion and promote venous metastasis in HCC [62], NK cells can directly kill tumor cells by releasing perforin and granzyme. TLR7/8 agonists can activate NK cells to suppress HepG2 xenograft growth, highlighting the therapeutic potential of NK cell modulation [63].

We used the CIBERSORT algorithm in R software to calculate the proportion of immune cells in the TME for each patient in the training and validation sets, and the TIDE algorithm to predict the efficacy of immunotherapy in the high-risk and low-risk groups. The results showed that in both the training and validation sets, Treg infiltration was increased in the high-risk group. Consistent results were observed in multiplex immunofluorescence experiments, showing more CD4^+^FoxP3^+^T cell infiltration in the high-risk group compared to the low-risk group. Additionally, the high-risk group had higher TIDE scores, increased immune exhaustion scores, and more MDSCs infiltration, suggesting a poorer response to immunotherapy. We also found that in the validation cohort, the majority of patients responding to immunotherapy were in the low-risk group, while the non-responders were predominantly in the high-risk group, indicating that low-risk patients were more sensitive to immunotherapy and had more NK cell infiltration. Studies have shown that increased lipid uptake and accumulation in the TME drive the TME toward an immunosuppressive phenotype that supports tumor progression [64]. Therefore, the poor prognosis and resistance to immunotherapy in high-risk patients may be due to enhanced de novo lipogenesis, which increases Treg cell function and impairs NK cell cytotoxicity in the TME. However, the underlying mechanisms require further exploration.

Although numerous biomarkers are used for HCC prognosis prediction, this study is the first to propose a prognostic model based on six genes related to de novo lipogenesis (G6PD, LCAT, SERPINE1, SOAT2, CYP2C9, and UGT1A10). We also collected a cohort of 106 patients from the Xiangya cohort to validate the model, demonstrating that it can predict HCC prognosis more effectively and reliably. Furthermore, we analyzed immune cell infiltration and treatment responses between high-risk and low-risk patients, providing a basis for personalized treatment strategies for HCC patients. Despite the clinical significance of our findings, there are some limitations. First, while incorporating real-world validation data, our study predominantly relied on public database analyses without experimental validation of the molecular mechanisms underlying prognostic associations. Second, the precise mechanisms through which differential immune microenvironment characteristics influence immunotherapy and targeted therapy responses remain to be fully elucidated. Finally, future studies should incorporate larger validation cohorts with more comprehensive clinicopathological data to strengthen the model’s generalizability.

## Conclusion

In our study, an effective method was provided to evaluate the prognostic value of de novo lipogenesis-related genes in HCC by constructing a novel prognostic risk prediction model including G6PD, LCAT, SERPINE1, SOAT2, CYP2C9, and UGT1A10. Additionally, we validated the expression levels of the model genes in cancerous and adjacent non-cancerous tissues, as well as their distribution among tumor microenvironment cell subpopulations. We then used multiplex immunofluorescence to assess differences in CD4^+^FoxP3^+^T cell infiltration between high-risk and low-risk patients. Moreover, TIDE scores combined with single-cell analysis and validation in targeted and immunotherapy cohorts further confirmed that high-risk patients are more suitable for targeted therapy, while low-risk patients respond better to immunotherapy. In conclusion, our research not only effectively predicts the progression and survival outcomes of HCC but also provides a theoretical foundation for formulating precise, personalized treatment strategies for HCC patients, offering a scientific basis for clinical decision-making.

## Electronic supplementary material

Below is the link to the electronic supplementary material.

Supplementary Material 1: Table S1. The list of de novo lipogenesis-related genes.

Supplementary Material 2: Table S2. The list of all DEGs in tumor and normal groups.

Supplementary Material 3: Table S3. The list of de novo lipogenesis-related DEGs

Supplementary Material 4: Table S4. GO terms in high and low risk groups.

Supplementary Material 5: Table S5. KEGG terms in high and low risk groups.

Supplementary Material 6: Table S6. The list of DEGs in high and low risk groups.

Supplementary Material 7: Fig. S1. Differential expression analysis and survival analysis of model genes in tumor and adjacent normal tissues in the TCGA-LIHC and Xiangya HCC cohorts. (A) Heatmap of differentially expressed genes in tumor and adjacent normal tissues in the TCGA-LIHC cohort. (B) Volcano plot of differentially expressed genes in the Xiangya HCC cohort. (C) Survival analyses of G6PD. (D)Survival analyses of SERPINE1. (E) Survival analyses of LCAT. (F) Survival analyses of CYP2C9. (G) Survival analyses of UGT1A10. (H) Survival analyses of SOAT2.

Supplementary Material 8: Fig. S2. (A)Kaplan–Meier curves of the OS of patients in the ICGC-LIRI training cohort. (B) ROC curves for predicting 1- and 3 year OS in the ICGC-LIRI training cohort. (C) Mutation type plots for the high-risk group. (D) Mutation type plots for low-risk group. (E) Ti/Tv ratio plots for the high-risk group. (F) Ti/Tv ratio plots for low-risk group. (G) TMB analysis for the high-risk and low-risk groups.

Supplementary Material 9: Fig. S3. Tumor Immune Microenvironment Analysis. (A) Correlation analysis of risk scores and model genes with each immune cell population in the Xiangya HCC cohort. (B) Immune cell infiltration analysis between high- and low-risk groups in the Xiangya HCC cohort. (C) Immune and stromal scores for high- and low-risk groups in the TCGA-LIHC cohort. (D) Immune and stromal scores for high- and low-risk groups in the Xiangya HCC cohort. (E) The box plot demonstrated the expression levels of TGF-β in the high-risk and low-risk groups.(F)The box plot demonstrated the expression levels of IL-10 in the high-risk and low-risk groups. (G) TIDE prediction of immunotherapy response in high- and low-risk groups in the TCGA-LIHC cohort. (H) TIDE prediction of immunotherapy response in high- and low-risk groups in the Xiangya HCC cohort. (I) Dysfunction scores for high- and low-risk groups in the TCGA-LIHC cohort. (J) Dysfunction scores for high- and low-risk groups in the Xiangya HCC cohort. * P < 0.05, ** P < 0.01, *** P < 0.001

Supplementary Material 10: Fig. S4. Relationship between Tumor Stage and Risk Score. (A) Comparison of risk scores between Stage I/II and Stage III/IV in the TCGA-LIHC cohort. (E) Comparison of risk scores between Stage I/II and Stage III/IV in the Xiangya HCC cohort. (B) Comparison of risk scores between T1/2 and T3/4 in the TCGA-LIHC cohort. (F) Comparison of risk scores between T1/2 and T3/4 in the Xiangya HCC cohort. (C) Comparison of risk scores between M stage M0 and M stage M1 in the TCGA-LIHC cohort. (D) Comparison of risk scores between N stage N0 and N stage N+ in the TCGA-LIHC cohort. ** P < 0.01

Supplementary Material 11: Fig. S5. Experimental Validation in the Xiangya HCC Cohort. (A)The SHAP plot illustrated the contributions of multiple genes to the model's predictive outcomes. (B) IHC chip showed G6PD expression in tumor tissues and paired adjacent tissues. (C) The expression of G6PD in normal liver cells and liver cancer cell lines

## Data Availability

All datasets presented in this study are included in the article/Supplementary Material. Further inquiries can be directed to the corresponding author.

## Abbreviations

HCC: Hepatocellular carcinoma
DNL: de novo lipogenesis
ScRNA: seq single-cell RNA sequencing
TMB: Tumor mutation burden
SREBPs: Sterol regulatory element-binding proteins
ACLY: ATP citrate lyase
ACAC: Acetyl-CoA carboxylase
FASN: Fatty acid synthase
SCD1: Stearoyl-CoA desaturase 1
DC: Dendritic cell
DEGs: Differentially expressed genes
K-M: Kaplan-Meier
C: Index consistency index
GO: Gene Ontology
KEGG: Kyoto Encyclopedia of Genes and Genomes
GSEA: Gene Set Enrichment Analysis
BP: Biological processes
CC: Cellular components
MF: Molecular functions
TIME: Tumor immune microenvironment
PPP: Pentose phosphate pathway
STCs: Senescent tumor cells
DFS: Disease-free survival
PFS: Progression-free survival
HDL: C high-density lipoprotein cholesterol
ECM: Extracellular matrix
UGT UDP: Glucuronosyltransferase
MDSCs Myeloid: derived suppressor cells
Tregs: Regulatory T cells
TME: Tumor microenvironment
