# Supplementary figures and images for: The impact of de novo lipogenesis on predicting survival and clinical therapy: an exploration based on a multigene prognostic model in hepatocellular carcinoma
Source: J Transl Med. 2025 Jun 18;23:679. doi: 10.1186/s12967-025-06704-y (PMC12178006; doi:10.1186/s12967-025-06704-y)

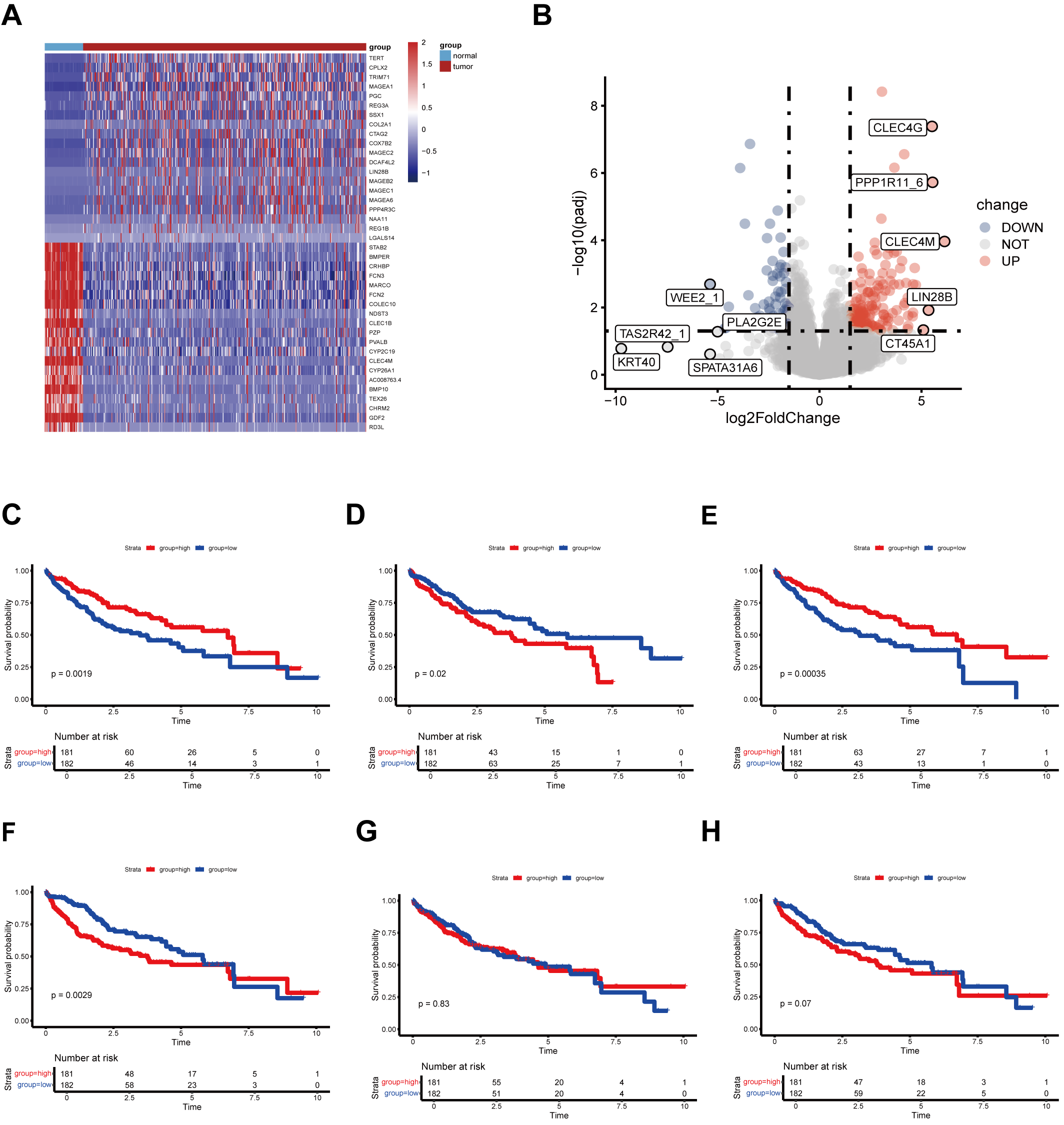

Supplement: Supplementary file 7 — Supplementary Material 7: Fig. S1. Differential expression analysis and survival analysis of model genes in tumor and adjacent normal tissues in the TCGA-LIHC and Xiangya HCC cohorts. (A) Heatmap of differentially expressed genes in tumor and adjacent normal tissues in the TCGA-LIHC cohort. (B) Volcano plot of differentially expressed genes in the Xiangya HCC cohort. (C) Survival analyses of G6PD. (D)Survival analyses of SERPINE1. (E) Survival analyses of LCAT. (F) Survival analyses of CYP2C9. (G) Survival analyses of UGT1A10. (H) Survival analyses of SOAT2. [file 12967_2025_6704_MOESM7_ESM.tif]

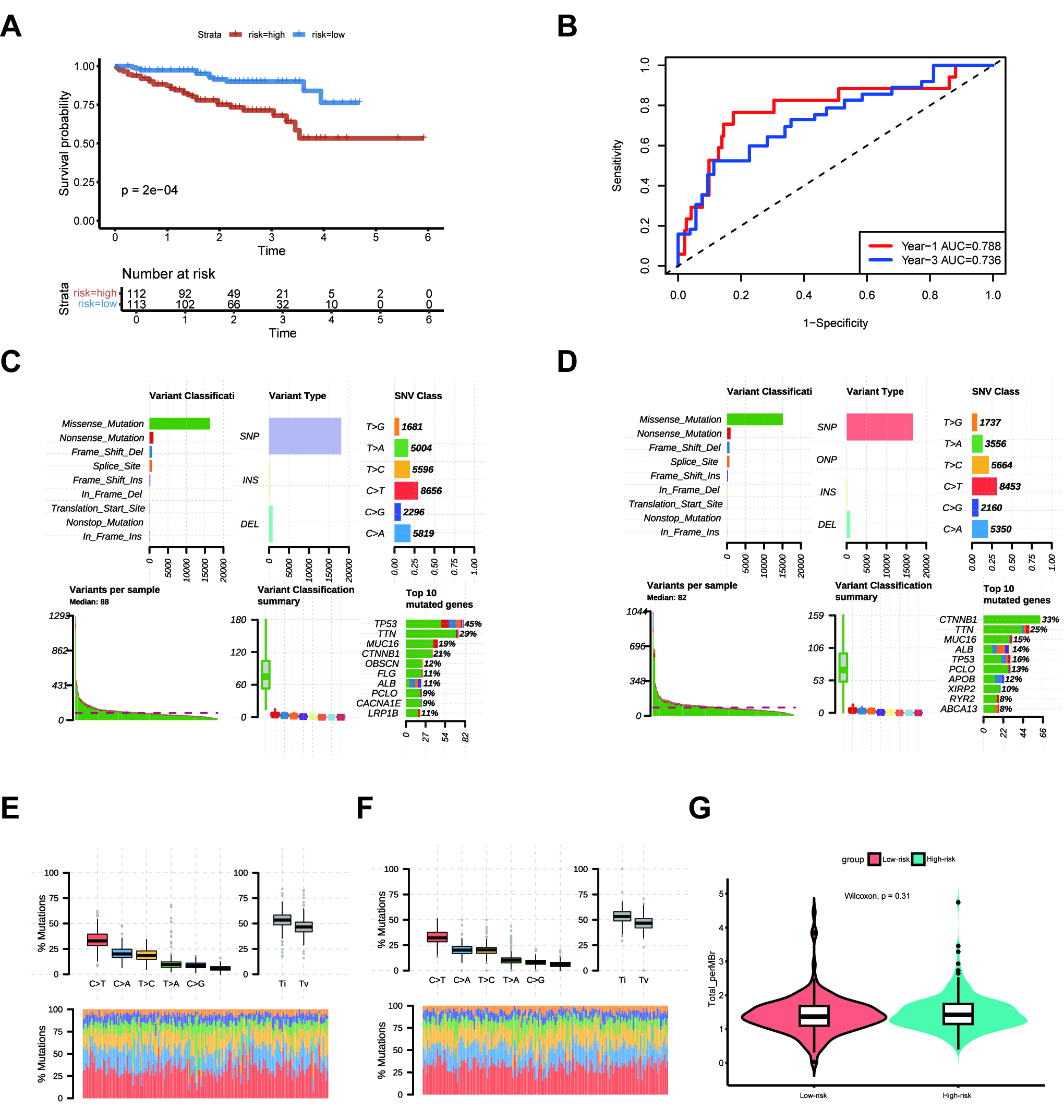

Supplement: Supplementary file 8 — Supplementary Material 8: Fig. S2. (A)Kaplan–Meier curves of the OS of patients in the ICGC-LIRI training cohort. (B) ROC curves for predicting 1- and 3 year OS in the ICGC-LIRI training cohort. (C) Mutation type plots for the high-risk group. (D) Mutation type plots for low-risk group. (E) Ti/Tv ratio plots for the high-risk group. (F) Ti/Tv ratio plots for low-risk group. (G) TMB analysis for the high-risk and low-risk groups. [file 12967_2025_6704_MOESM8_ESM.tif]

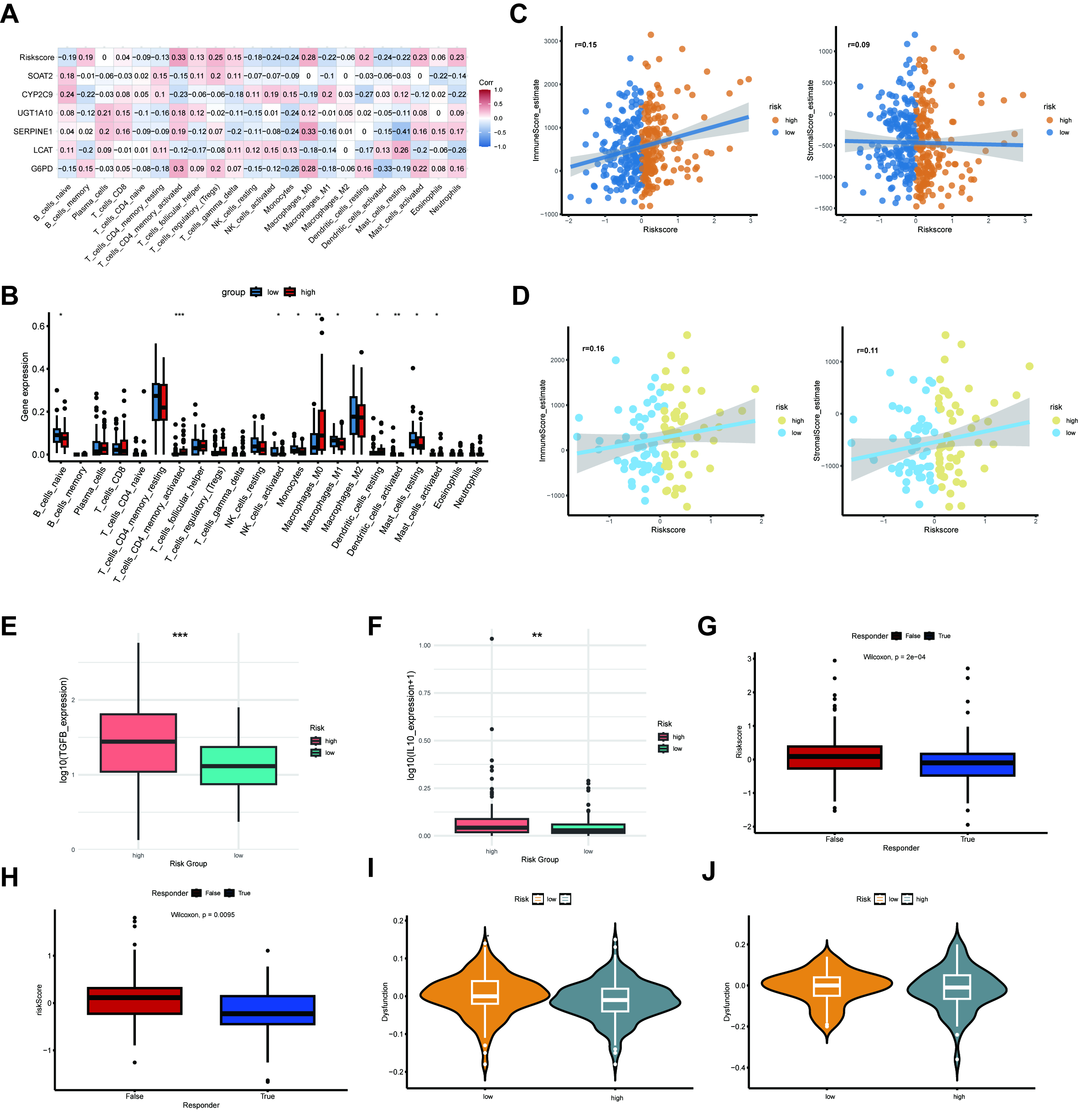

Supplement: Supplementary file 9 — Supplementary Material 9: Fig. S3. Tumor Immune Microenvironment Analysis. (A) Correlation analysis of risk scores and model genes with each immune cell population in the Xiangya HCC cohort. (B) Immune cell infiltration analysis between high- and low-risk groups in the Xiangya HCC cohort. (C) Immune and stromal scores for high- and low-risk groups in the TCGA-LIHC cohort. (D) Immune and stromal scores for high- and low-risk groups in the Xiangya HCC cohort. (E) The box plot demonstrated the expression levels of TGF-β in the high-risk and low-risk groups.(F)The box plot demonstrated the expression levels of IL-10 in the high-risk and low-risk groups. (G) TIDE prediction of immunotherapy response in high- and low-risk groups in the TCGA-LIHC cohort. (H) TIDE prediction of immunotherapy response in high- and low-risk groups in the Xiangya HCC cohort. (I) Dysfunction scores for high- and low-risk groups in the TCGA-LIHC cohort. (J) Dysfunction scores for high- and low-risk groups in the Xiangya HCC cohort. * P < 0.05, ** P < 0.01, *** P < 0.001 [file 12967_2025_6704_MOESM9_ESM.tif]

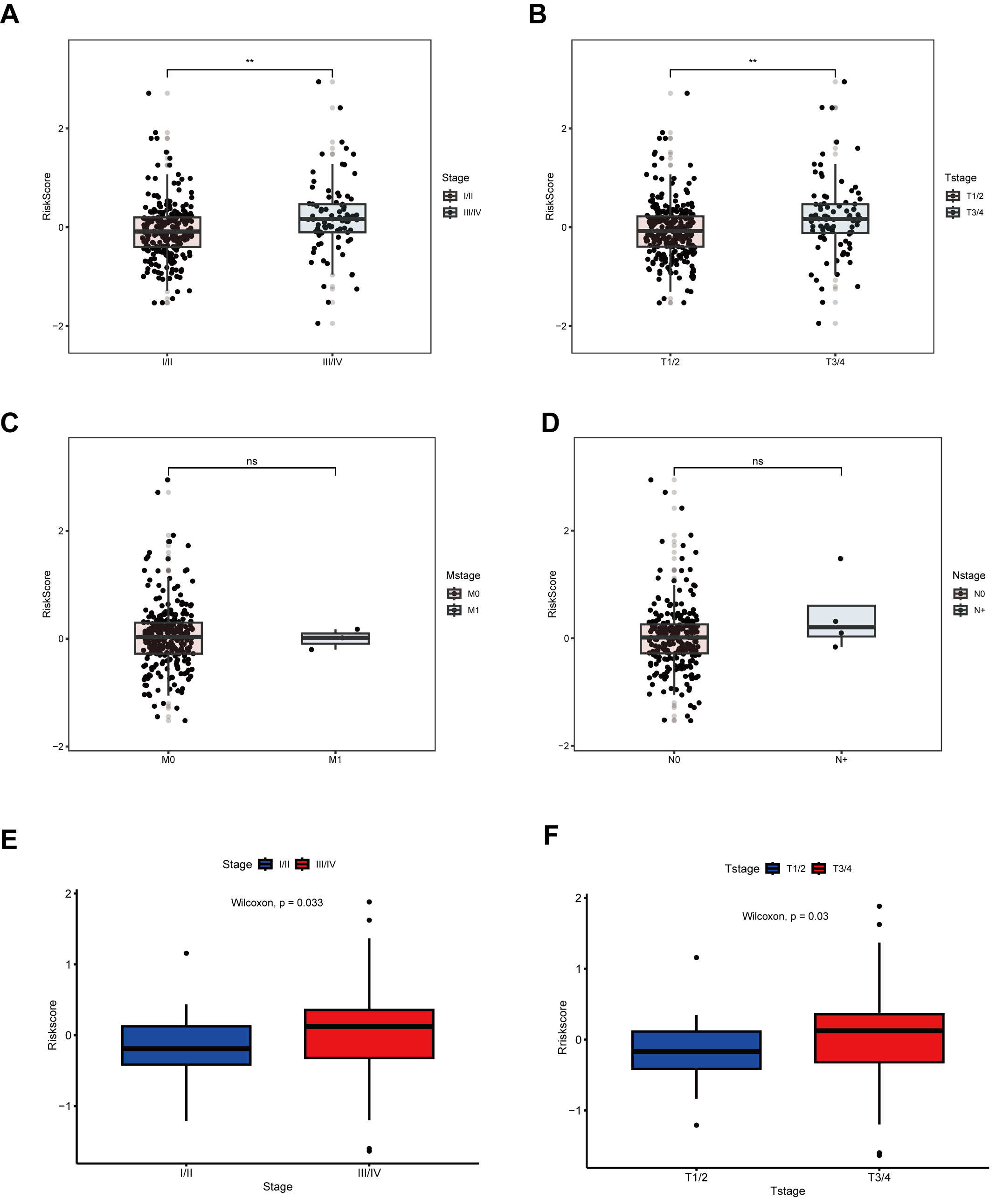

Supplement: Supplementary file 10 — Supplementary Material 10: Fig. S4. Relationship between Tumor Stage and Risk Score. (A) Comparison of risk scores between Stage I/II and Stage III/IV in the TCGA-LIHC cohort. (E) Comparison of risk scores between Stage I/II and Stage III/IV in the Xiangya HCC cohort. (B) Comparison of risk scores between T1/2 and T3/4 in the TCGA-LIHC cohort. (F) Comparison of risk scores between T1/2 and T3/4 in the Xiangya HCC cohort. (C) Comparison of risk scores between M stage M0 and M stage M1 in the TCGA-LIHC cohort. (D) Comparison of risk scores between N stage N0 and N stage N+ in the TCGA-LIHC cohort. ** P < 0.01 [file 12967_2025_6704_MOESM10_ESM.tif]

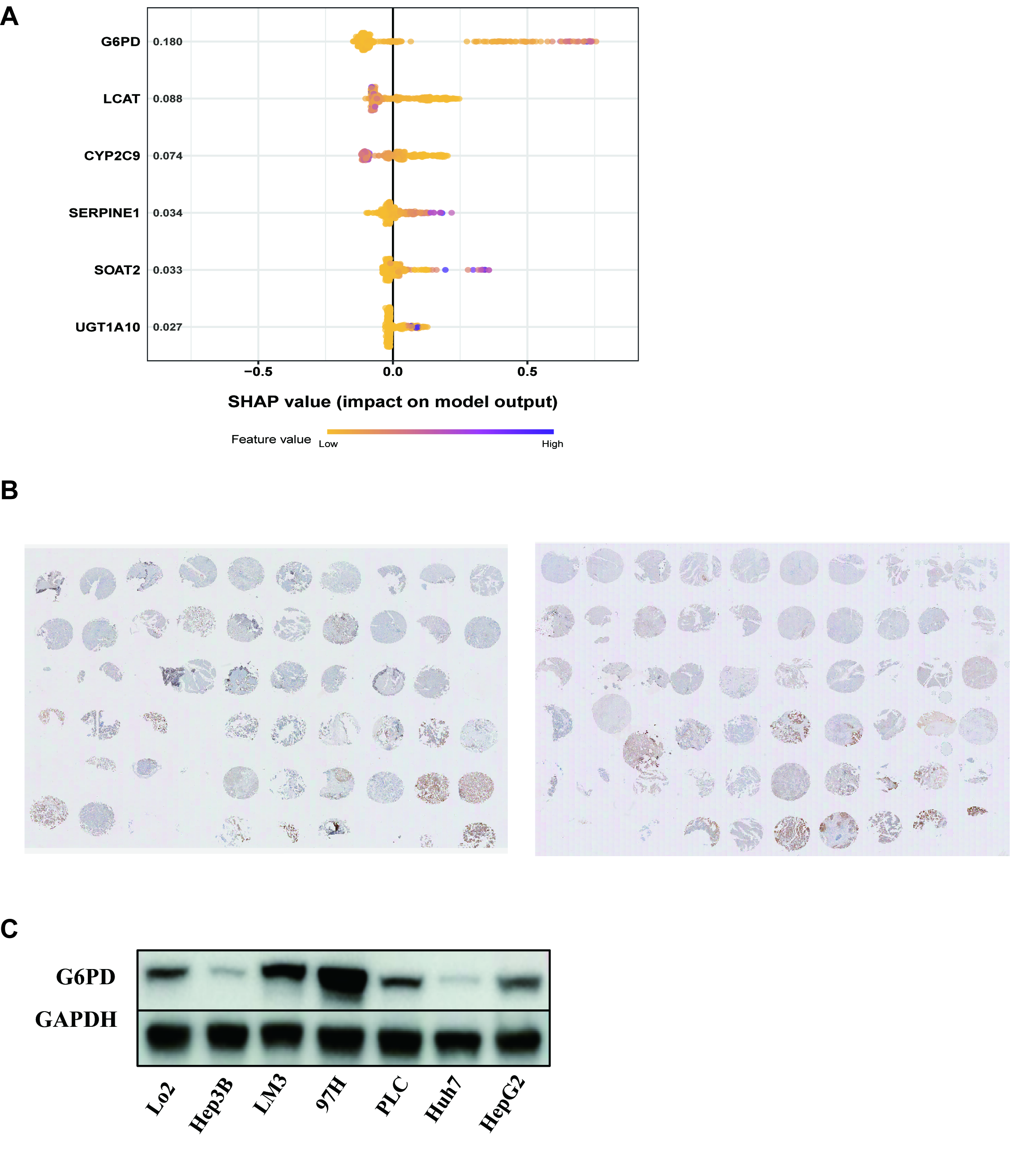

Supplement: Supplementary file 11 — Supplementary Material 11: Fig. S5. Experimental Validation in the Xiangya HCC Cohort. (A)The SHAP plot illustrated the contributions of multiple genes to the model's predictive outcomes. (B) IHC chip showed G6PD expression in tumor tissues and paired adjacent tissues. (C) The expression of G6PD in normal liver cells and liver cancer cell lines [file 12967_2025_6704_MOESM11_ESM.tif]
